# Filopodial protrusion driven by density-dependent Ena–TOCA-1 interactions

**DOI:** 10.1242/jcs.261057

**Published:** 2024-03-21

**Authors:** Thomas C. A. Blake, Helen M. Fox, Vasja Urbančič, Roshan Ravishankar, Adam Wolowczyk, Edward S. Allgeyer, Julia Mason, Gaudenz Danuser, Jennifer L. Gallop

**Affiliations:** ^1^ Wellcome/Cancer Research UK Gurdon Institute, University of Cambridge, Cambridge CB2 1QN, UK; ^2^ Department of Biochemistry, University of Cambridge, Cambridge CB2 1QW, UK; ^3^ Lyda Hill Department of Bioinformatics, UT Southwestern Medical Center, Dallas, TX 75390, USA; ^4^ Department of Genetics, University of Cambridge, Cambridge CB2 3EH, UK

**Keywords:** Growth cone, Actin, Migration

## Abstract

Filopodia are narrow actin-rich protrusions with important roles in neuronal development where membrane-binding adaptor proteins, such as I-BAR- and F-BAR-domain-containing proteins, have emerged as upstream regulators that link membrane interactions to actin regulators such as formins and proteins of the Ena/VASP family. Both the adaptors and their binding partners are part of diverse and redundant protein networks that can functionally compensate for each other. To explore the significance of the F-BAR domain-containing neuronal membrane adaptor TOCA-1 (also known as FNBP1L) in filopodia we performed a quantitative analysis of TOCA-1 and filopodial dynamics in *Xenopus* retinal ganglion cells, where Ena/VASP proteins have a native role in filopodial extension. Increasing the density of TOCA-1 enhances Ena/VASP protein binding *in vitro*, and an accumulation of TOCA-1, as well as its coincidence with Ena, correlates with filopodial protrusion *in vivo*. Two-colour single-molecule localisation microscopy of TOCA-1 and Ena supports their nanoscale association. TOCA-1 clusters promote filopodial protrusion and this depends on a functional TOCA-1 SH3 domain and activation of Cdc42, which we perturbed using the small-molecule inhibitor CASIN. We propose that TOCA-1 clusters act independently of membrane curvature to recruit and promote Ena activity for filopodial protrusion.

## Teaser

**Summary:** Quantitative dynamic analysis of neuronal growth cone filopodia formation reveals a density-dependent interaction between the membrane adaptor TOCA-1 and actin filament elongating protein Ena.

## INTRODUCTION

Axonal growth cone navigation underlies accurate neuronal connectivity in the brain and is guided by chemical and mechanical cues that are transduced to the cytoskeletal machinery to enable movement and turning (O'Connor et al., 1990; Koser et al., 2016). A key element in this navigation process is the protrusion of filopodia through the dynamic growth and shrinkage of long, unbranched bundles of actin filaments, controlled by actin regulators at the filopodium tip (Mallavarapu and Mitchison, 1999; Applewhite et al., 2007; Blake and Gallop, 2023). In the axonal growth cone, stochastic protrusion of filopodia has roles in transient adhesion to surfaces and promotion of accurate movement (O'Connor et al., 1990; Dwivedy et al., 2007). Control of filopodial protrusion, retraction and adhesion allows cells to transduce signals and transmit force to the environment via an adhesion-based molecular clutch (Chan and Odde, 2008; He et al., 2017). The actin bundle is surrounded by plasma membrane with high negative curvature that is stabilised by proteins that link curved plasma membrane to the cytoskeleton, such as IRSp53 (also known as BAIAP2) (Nakagawa et al., 2003; Mattila et al., 2007; Tsai et al., 2018). How the process of filopodial protrusion is controlled is important for understanding the mechanistic and genetic bases of intellectual disabilities and autism, amongst other conditions (Truesdell et al., 2015; Hu et al., 2016; Gouder et al., 2019; Wit and Hiesinger, 2022). As an established axon guidance model, here we used *ex vivo* dissected primary *Xenopus* retinal ganglion cells (RGCs) to elucidate the mechanisms of filopodial extension. In embryos, the growth cones of these cells migrate from eye primordia along a laminin matrix to the tectum in the brain, and when explanted onto laminin-coated dishes, they migrate with large growth cones and are amenable to imaging with high temporal and spatial resolution (Koser et al., 2016; Urbančič et al., 2017).

Many actin regulatory proteins that contribute to filopodial growth, including proteins of the Ena/VASP family and myosin-X, have been shown to localise to the tips of filopodia, and their depletion or knockout alters the number or length of filopodia, revealing that they play a direct or indirect functional role (Lebrand et al., 2004; Barzik et al., 2014; Jacquemet et al., 2019; Pokrant et al., 2023). Initiation pathways of filopodia have been proposed based on recruitment of the membrane adaptor protein IRSp53, which deforms the membrane and in turn recruits VASP to elongate actin filaments (Disanza et al., 2013; Sudhaharan et al., 2019; Tsai et al., 2022). Dynamic complexes of another initiating membrane adaptor, lamellipodin (also known as RAPH1), with VASP have also been shown to grow to a defined stoichiometry for controlled and productive protrusion (Cheng and Mullins, 2020). Ena/VASP family proteins determine force generation and actin architecture in lamellipodia (Bear et al., 2002; Damiano-Guercio et al., 2020) and are localised to the growing tips of filopodia, where their presence correlates with new actin monomers being incorporated into the elongating bundle (Applewhite et al., 2007; Urbančič et al., 2017). Biochemically, Ena and VASP are processive actin-elongating proteins requiring G-actin-binding activity, F-actin-binding activity and oligomerisation for their function (Breitsprecher et al., 2008; Hansen and Mullins, 2015; Harker et al., 2019) and are regulated by post-translational modifications including ubiquitylation (McCormick et al., 2024).

It remains unclear how interaction partners and mechanisms combine to control Ena and VASP activation, since neither IRSp53 nor lamellipodin are essential for Ena or VASP recruitment (Pokrant et al., 2023). What triggers filopodial formation at certain sites and controls when filopodia grow or retract is unknown. Other candidate membrane adaptors that localise to filopodia are members of the neuronally enriched TOCA-1 (also known as FNBP1L) family, which includes FBP17 (also known as FNBP1) and paralogue CIP4 (also known as TRIP10; present in mammals but not in *Xenopus*). TOCA-1 family proteins promote lamellipodial, filopodial and neurite formation in conjunction with GTP-bound Cdc42, N-WASP (also known as WASL) and WAVE complex (Ho et al., 2004; Kakimoto et al., 2006; Bu et al., 2009; Fricke et al., 2009; Hu et al., 2011; Saengsawang et al., 2012, 2013). TOCA-1 has previously been studied in EGFR-activated A431 epidermoid carcinoma cells, where it has EGFR-dependent roles in filopodia, endocytosis and cell motility (Hu et al., 2011). In mouse cortical neurons, TOCA-1 family members each promote or oppose neurite formation and endocytosis, depending on the isoform expressed (Taylor et al., 2019). In these neurons, but not in COS7 cells, CIP4 localises to the tips of extending filopodia and is proposed to interact with the Ena protein Mena (also known as Enah) as well as with the formin DAAM1, phosphoinositide lipids and uncapped actin to promote the formation of bundled actin structures in axonal growth cones (Saengsawang et al., 2012, 2013).

There is conflicting evidence about whether TOCA-1 contributes positively or negatively to filopodial protrusion: TOCA-1 overexpression has been found to induce filopodial formation (Bu et al., 2009), whereas TOCA-1 knockdown or CIP4 knockout results in increased numbers of filopodia (Hu et al., 2011; Saengsawang et al., 2012). Because of their positively curved F-BAR domains, TOCA-1 family proteins have been implicated more in endocytic scenarios (Tsujita et al., 2006; Bu et al., 2009; Giuliani et al., 2009; Feng et al., 2010; Ledoux et al., 2023), and any role at the tips of filopodia would seem inconsistent with the proposed curvature sensitivity.

Distinguishing between different mechanistic functions suggested by genetic or chemical perturbation can be complex. In filopodia, upregulation of the formins FMNL2 and FMNL3 takes place after acute removal of the Arp2/3 complex member Arp3 (ACTR3), inducing filopodial formation (Dimchev et al., 2021). Conversely, IRSp53 and other I-BAR domain-containing proteins can generate filopodia but are not essential (Pokrant et al., 2023). Altering the level of G-actin-sequestering protein profilin 1 to different extents leads to mass changes in cellular filopodial or lamellipodial architecture, distinct from a model where proteins are limited to specific roles in generating individual filopodia (Skruber et al., 2020). As well as competition between the distinct, interconnected F-actin networks in the cell for actin monomers (Burke et al., 2014; Dimchev et al., 2017; Kadzik et al., 2020), redundancy and stochasticity in regulatory protein composition and dynamics have themselves been implicated as key underlying mechanisms in filopodial generation (Dobramysl et al., 2021; Mancinelli et al., 2021).

Quantitative image analysis employing cross-correlation analysis and fluctuation analysis of protein recruitment alongside morphological changes offers the opportunity to: (1) investigate a protein within its physiologically relevant spatio-temporal context and interaction network; (2) analyse ‘non-essential’ proteins (such as TOCA-1), where knockdown or knockout approaches do not reveal a decisive phenotype; and (3) where a protein has multiple proposed functions from perturbation experiments, to study each proposed function individually and distinguish scenarios where the protein has a functional role distinct from localisation alone (Welf and Danuser, 2014; Lee et al., 2015). Recent work analysing the regulation of actin dynamics in lamellipodia demonstrates how Granger causality analysis (GCA) can extract bona fide cause–effect relations from imaged time series, distinguishing between proteins that drive lamellipodial extension and proteins that are present but do not functionally contribute, such as a polymerisation-deficient VASP mutant (Noh et al., 2022).

Filopodia represent a powerful system for employing these methods because filopodial protrusion is a fast-moving morphological readout for dynamic changes in protein recruitment, and their shape means that they are an easily definable object to analyse. In previous work we used cross-correlation analysis to demonstrate that the arrival of Ena and VASP at filopodia precedes future protrusion in a significant subset of filopodia, recapitulating insights from knockout studies for Ena and VASP in the absence of genetic perturbation (Urbančič et al., 2017). Here, to address the role of the F-BAR domain protein TOCA-1 in filopodial formation we have used a combination of *in vitro* binding assays, cross-correlation analysis and GCA of filopodial dynamics, as well as perturbation of Cdc42 using small-molecule inhibitor treatment, to demonstrate the contribution of TOCA-1 to Ena recruitment and filopodial extension. We show that the coincidence of TOCA-1 and Ena at lamellipodia and the tips of filopodia precedes forward filopodial movement in native filopodia and propose a density-dependent switch in TOCA-1 binding to Ena at the start of cycles of filopodial protrusion.

## RESULTS

### TOCA-1 interacts with Ena and VASP in a density-dependent manner and localises to filopodia

**Fig. 1.**
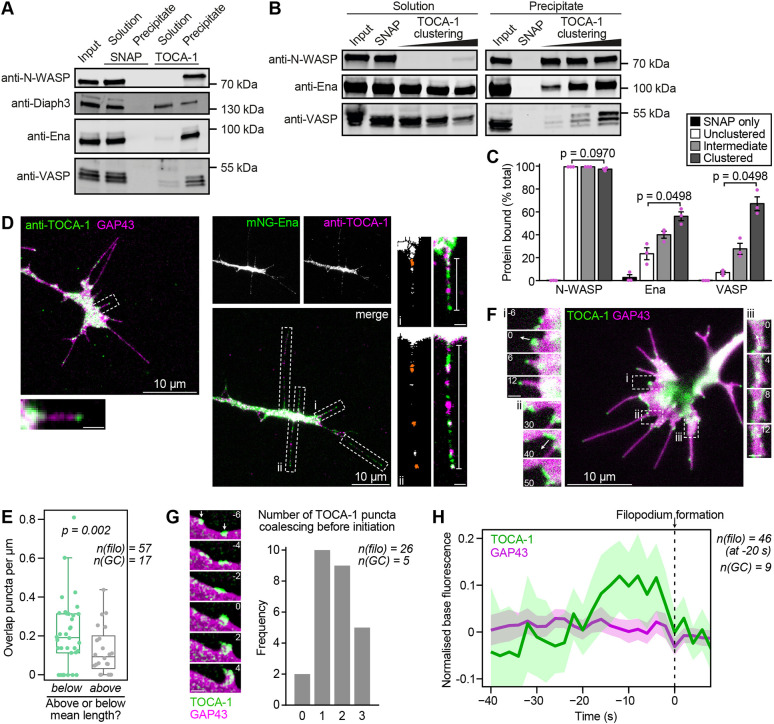
**TOCA-1 interacts with Ena and VASP and localises to filopodia.** (A) SNAP alone or SNAP–TOCA-1 coupled to beads and incubated with *Xenopus* egg HSS extract, with western blotting for the indicated known and candidate TOCA-1-interacting proteins. None of the proteins bound to SNAP alone, remaining in the solution fraction, whereas all were bound by SNAP–TOCA-1 and were detected in the precipitate fraction. (B) SNAP–TOCA-1 (1 nmol) was coupled to 10, 50 or 100 µl beads (clustered, intermediate and unclustered, shown in lanes five, four and three, respectively) followed by addition of beads up to a volume of 100 µl and an excess of SNAP protein in a second coupling step. After incubation with HSS (containing ∼90 pmol Ena, 30 pmol each VASP and N-WASP) and separation into unbound (solution) and bound (precipitate) fractions, Ena and VASP are found to bind more efficiently at higher densities of TOCA-1, whereas N-WASP binding efficiency is unaffected. A bead sample coupled to SNAP alone is shown as a binding control. (C) Quantification of protein bound in B (as a percentage of the sum intensity of precipitate and solution bands; mean±s.e.m., *n*=3). *P*-values on the graph are from a Friedman test for all bound conditions of each protein group. Nemenyi post hoc tests confirm that Ena and VASP had significant differences between unclustered and clustered states (both *P*=0.038; other pairwise comparisons not significant). (D) Left: a fixed RGC expressing GAP43–RFP, immunostained with affinity-purified anti-TOCA-1, showing puncta of TOCA-1 in shafts and tips of filopodia (see example indicated by the dashed box and shown enlarged beneath the main image). Right: a fixed RGC expressing mNG–Ena and immunostained to detect TOCA-1 show that both proteins form multiple, distinct puncta along filopodia, sometimes at bases (i) and shafts, and sometimes at tips (ii). White dashed boxes indicate filopodia subject to manual quantification; enlarged views of examples i and ii are shown beside the main image alongside thresholded images with regions of overlapping TOCA-1 and Ena in orange. Brackets indicate filopodium length. The number of overlapping puncta is ∼30% of either Ena or TOCA-1 puncta numbers. (E) Grouping filopodia by size reveals that shorter filopodia have a higher density of TOCA-1 and Ena overlap puncta (*P*=0.002; Kruskal–Wallis test). Box plot shows the median (line), interquartile range (box) and range (whiskers), excluding outliers (more than 1.5× the IQR outside the upper or lower quartiles). (F) A *Xenopus* RGC axonal growth cone (Movie 1) after electroporation with mNG–TOCA-1 and the membrane-binding region of GAP43–RFP, showing localisation of mNG–TOCA-1 to (i) filopodia, (ii) advancing lamellipodia and (iii) inwardly-moving puncta in the growth cone body. Enlarged images show montages of the regions marked by dashed boxes. Arrows show the direction of punctum movement. Images are representative of 11 growth cones. (G) Left: example montage (Movie 2) shown with two distinct puncta indicated by arrows. Images de-noised with nd-safir. Right: scoring of the number of distinct puncta of mNG–TOCA-1 observed to coalesce in the preceding few seconds at the site of future filopodium formation. (H) Measuring fluorescence intensity of mNG–TOCA-1 and GAP43–RFP at the predicted base before filopodium formation shows that mNG–TOCA-1 fluorescence peaks at ∼10 s prior to filopodium emergence (dashed line) and reduces before protrusion begins. Lines indicate mean values; shading represents 95% c.i. All scale bars are 1 µm unless indicated otherwise. Time is shown in seconds relative to filopodium formation. *n*(filo), number of filopodia; *n*(GC), number of growth cones.

Within a cell-free model of filopodia-like structures that uses *Xenopus* egg extracts applied to supported lipid bilayers to produce fascin-bundled actin structures (Lee et al., 2010), the abundance of TOCA-1 has a notable correlation with the abundance of Ena and VASP at sites of actin incorporation (Dobramysl et al., 2021). To determine whether TOCA-1 is capable of interaction with Ena and VASP directly, we covalently coupled recombinant SNAP-tagged *Xenopus tropicalis* TOCA-1, or the SNAP tag alone, to benzylguanine-derivatised magnetic beads and incubated them with *Xenopus* egg high-speed supernatant (HSS) extracts. SNAP–TOCA-1 beads precipitated the known interaction partners N-WASP and Diaph3, plus Ena and VASP (Fig. 1A).

The TOCA-1 family of proteins forms both dimers and higher-order oligomers on membranes, where it clusters monomeric N-WASP via its SH3 domain interaction (Frost et al., 2008; Padrick and Rosen, 2010). Ena and VASP contain a tetramerisation domain, and their increased clustering scales with their processivity of actin filament elongation (Breitsprecher et al., 2008; Harker et al., 2019). Therefore, we tested whether the interaction of TOCA-1 with N-WASP, Ena or VASP differs in response to the level of clustering of TOCA-1 (Fig. 1B). We mimicked the effect of TOCA-1 clustering on membranes by tuning the density of SNAP–TOCA-1 on beads and incubating them with extract to mimic cytosol; under these conditions the concentrations were 71 nM N-WASP, 70 nM VASP and 132 nM Ena (Dobramysl et al., 2021). We used 1 nmol SNAP–TOCA-1 at different densities on the beads in a 0.4 ml assay volume, resulting in a 10–30-fold excess of TOCA-1 binding sites to any single binding partner (each present at ∼30–90 pmol). Whereas all the N-WASP was bound by a fixed quantity of TOCA-1 regardless of whether it was sparsely or densely coupled to the beads, Ena and VASP showed a strong preference for a dense coupling of TOCA-1 (Fig. 1C), suggesting that in cells with the array of binding partners, Ena and VASP might interact dynamically with TOCA-1 only when it reaches high density.

TOCA-1 has previously been seen to localise to and stimulate filopodia in N1E-115 neuroblastoma cells and mouse cortical neurons (Bu et al., 2009; Saengsawang et al., 2012). To verify that endogenous TOCA-1 localises to natively forming filopodia in our system we performed immunostaining of *Xenopus* RGC axonal growth cones, finding that endogenous TOCA-1 localises to the tips and shafts of filopodia (Fig. 1D; Fig. S1). In RGCs expressing mNeonGreen (mNG)-tagged Ena (mNG–Ena), immunostaining of endogenous TOCA-1 revealed that, while TOCA-1 and Ena puncta were often distinct, many filopodia shafts and tips contained both TOCA-1 and Ena (Fig. 1D). We quantified the areas of overlap, finding typically one to three areas per filopodium and a higher frequency of overlap puncta in shorter filopodia (Fig. 1E). To quantify the dynamics of TOCA-1 recruitment to filopodia, we expressed mNG–TOCA-1 and the membrane marker GAP43–RFP in RGCs, revealing the dynamics of TOCA-1 at filopodia tips, advancing lamellipodia and inwardly-moving puncta within the central domain (Fig. 1F; Movie 1). Across all filopodia at any given timepoint, 64% of filopodia had TOCA-1 present in the shaft or tip (mean of three frames, from 11 growth cones with 53 filopodia on average). Among newly forming filopodia, 65% had TOCA-1 at the tip. In 41% of filopodia, TOCA-1 could be seen departing from the base of the filopodium during formation (17 filopodia from five growth cones), similar to the splitting of filopodial initiation complexes implicated in maintaining lamellipodin–VASP stoichiometry (Cheng and Mullins, 2020). Tracking individual TOCA-1 plasma membrane-localised puncta over 4 min time-lapse videos revealed that 46% of puncta persisted at the plasma membrane not at a filopodium, 34% moved inwardly to the central domain and 21% localised to filopodia, either at the tips or mobile within the shaft (107 TOCA-1 puncta from four growth cones).

Given that the positive curvature sensing of the TOCA-1 F-BAR domain would seemingly be in opposition to the curvature of filopodia tips, we wanted to determine whether the localisation of TOCA-1 to filopodia tips was functionally significant. Inspection of the lamellipodial TOCA-1 puncta that went on to form filopodia revealed they increased in fluorescence density in the few seconds before filopodium formation. In 14 of 26 filopodia, two or three distinct puncta of TOCA-1 coalesced in the few seconds before formation, creating a single bright punctum that usually stayed at the tip (Fig. 1G; Movie 2). Across all formation events, TOCA-1 fluorescence intensity at the site of formation increased in the 10–20 s prior to protrusion and then decreased before tip emergence (−5 s to −2 s timepoints relative to emergence) (Fig. 1H). This is earlier than Ena accumulation, which peaks at 2 s prior to protrusion and only drops as the tip emerges (Urbančič et al., 2017), suggesting that dense TOCA-1 transiently accumulates at the initiation of filopodia, where it could interact with Ena.

### Quantification of TOCA-1 fluorescence and filopodial protrusion demonstrates that TOCA-1 and Ena have similar relationships to filopodial movement

Having established that TOCA-1 and Ena/VASP proteins undergo a density-dependent interaction *in vitro* and partially overlap in cells, we analysed filopodia from 11 growth cones expressing mNG–TOCA-1 using our semi-automated analysis pipeline, Filopodyan, to compare the behaviour of TOCA-1 to Ena or VASP in filopodial extension and other dynamic behaviours. In our previous analysis of Ena and VASP in ongoing filopodial protrusion we used cross-correlation analysis to identify subpopulations of Ena- and VASP-responding filopodia, showing that Ena and VASP fluorescence at filopodia tips correlates with tip extension (Urbančič et al., 2017).

**Fig. 2.**
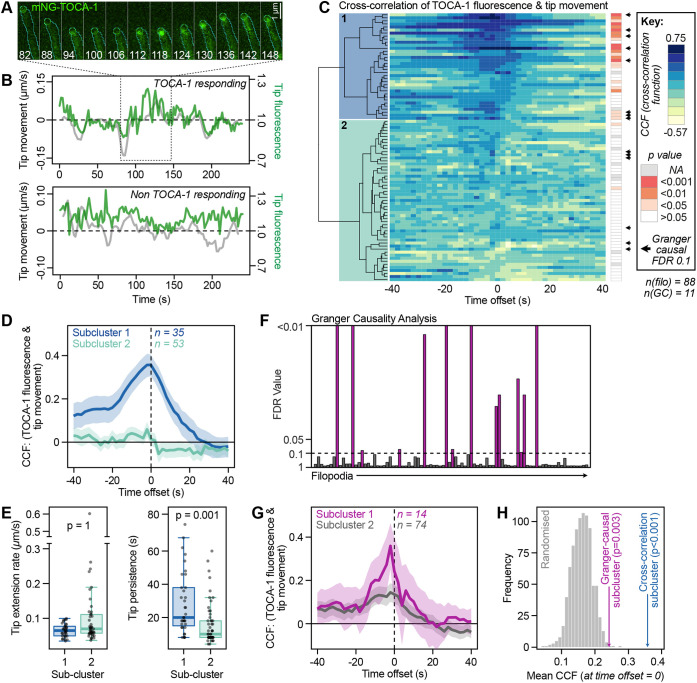
**TOCA-1 has a functional role during filopodial tip extension.** (A) Montage of a filopodium (cyan outline) and tip (green circle) segmented by Filopodyan, showing an increase in mNG–TOCA-1 fluorescence at the moment of forward tip movement. Time is indicated in seconds. (B) Quantification of mNG–TOCA-1 fluorescence intensity at the tip and tip movement of two independent filopodia with distinct behaviours. Data corresponding to the TOCA-1-responding filopodium in A are highlighted by a dashed box. (C) Cross-correlation function (CCF) scores for each filopodium at multiple time offsets between tip movement and tip TOCA-1 fluorescence, with hierarchical clustering based on CCF scores from −6 s to +6 s. The two subclusters identified are indicated on the dendrogram in blue and green. Colour shows degree of correlation. Likelihood of the observed level of correlation occurring by chance in individual filopodia is indicated by grey and orange boxes to the right, as computed using Markov Chain simulations. *P*-values shown in the figure are not corrected for multiple comparisons (NA, not available). Filopodia with a Granger-causal relationship between TOCA-1 fluorescence and tip movement are marked by arrows. *n*(filo), number of filopodia; *n*(GC), number of growth cones. (D) Averaging the top-correlating ‘TOCA-1-responding’ subcluster (subcluster 1 in C, blue) separately from the rest of the filopodia (subcluster 2 in C, green) reveals a strong correlation between tip movement and TOCA-1 fluorescence, peaking at −2 s time offset. Lines indicate mean values; shading represents 95% c.i. (E) TOCA-1-responding filopodia (subcluster 1) have no change in tip extension rate (left), whereas persistence of tip movement (right; based on the auto-correlation function) is increased twofold. Box plots as in Fig. 1E. *n* values as in D. Significance assessed with Mann–Whitney tests, with multiple comparisons corrected using the Holm method. (F) GCA confirms that some of the TOCA-1-responding filopodia are Granger causal for tip movement (Benjamini–Hochberg-corrected FDR<0.1; black arrows in C). (G) A TOCA-1-responding subcluster defined using the GCA criterion (subcluster 1) still has greater mean CCF score than the remaining filopodia (subcluster 2). Lines indicate mean values; shading represents 95% c.i. (H) A randomisation approach to testing significance shows that the observed degree of correlation in the TOCA-1-responding subclusters defined by cross-correlation (blue arrow) or GCA (purple arrow) is stronger than would be expected if TOCA-1 tip fluorescence and tip movement were decoupled, since out of 1000 reshuffled datasets, 0 and 3, respectively, produced a subcluster with higher mean CCF at *t*=0, with *P*-values calculated as fraction of reshuffled datasets exceeding observed.

By measuring tip movement speed alongside tip mNG–TOCA-1 fluorescence at each timepoint (Fig. 2A,B), we calculated a cross-correlation function (CCF) score for each filopodium, scoring from +1 (perfect positive correlation) to −1 (perfect negative correlation). To identify any offset in peak tip fluorescence relative to tip movement, CCFs were calculated across a range of time offsets, from −40 s to +40 s, followed by hierarchical clustering of the filopodia by their CCF scores (Fig. 2C). A continuum was seen between weak negative correlation, weak positive correlation and, for a subcluster formed by 35 ‘TOCA-1-responding’ filopodia (from a total of 88 quantified), strong positive correlation, similar to our previous findings with Ena and VASP. For most of the TOCA-1-responding filopodia, the CCF scores were significantly higher than expected by chance, as tested with a Markov chain-based simulation (simulated CCFs exceeded the observed values in fewer than 500 of 10,000 cases for 23 of the 35 filopodia in subcluster 1; Fig. 2C). RGCs expressing mNG–TOCA-1 had no significant differences in any of the measured dynamic parameters, as compared to RGCs expressing mNG alone (Fig. S2, Table S1).

On average, the TOCA-1-responding filopodia had peak correlation between TOCA-1 tip fluorescence and tip movement at an offset of 2 s prior to forward tip movement (−2 s offset; mean CCF=0.36; Fig. 2D). This difference in CCF score between the subclusters was robust to different dividing points (Fig. S3A). Exploring the dynamic properties of the two subclusters revealed that TOCA-1-responding filopodia had a twofold increase in the persistence of tip movement (Fig. 2E; Table S2), and this increase was robust to variation of the subclustering point or variation of data processing parameters such as smoothing (Fig. S3B,C). Like Ena, and in contrast to VASP (Urbančič et al., 2017), the TOCA-1-responding subcluster had no significant change in tip extension rate (Fig. 2E), suggesting a specific function for TOCA-1 in facilitating persistent filopodial growth.

### Granger causality analysis validates the TOCA-1-responding subpopulation identified using cross-correlation analysis

Correlation between protein recruitment to dynamic regions of the cell and cell morphology does not always imply functional relevance at that site. Therefore, to distinguish between situations where TOCA-1 is ‘passively’ localising to extending filopodia tips and TOCA-1 having an active functional role, we used GCA, which tests whether fluctuations in a given signal (here, TOCA-1 tip fluorescence) are essential for predicting future fluctuations in another signal (tip movement). This approach infers the level of causality and the hierarchy of cause and effect between two variables (Noh et al., 2022).

In essence, GCA tests whether the past values of variable *A* are informative in explaining the future values of a variable *B*. To do so, the procedure relies on two regression models. The first model (the reduced model) describes the value of variable *B* at time *t*, *B_t_*, as a function of the previous *p* values of *B*. The second model (the full model) also considers the past *r* values of *A*. The optimal lag orders *p* and *r* are determined by model selection using the Bayesian information criterion. The variable *A* is deemed causal for *B* if the variance of the residual variable of the second model is significantly reduced compared the variance of the residual variable of the first model. Applying this approach to the same time series of TOCA-1 tip fluorescence as variable *A* and filopodia tip movement as variable *B*, we found a subset of filopodia (14 of 88) in which TOCA-1 fluorescence was Granger causal for tip movement at a false discovery rate (FDR) of 0.1 (Fig. 2F).

This subset overlapped with the subset of TOCA-1-responding filopodia defined by CCF scores (Fig. 2C, black arrows) confirming that both methods identify similar filopodia with functionally relevant TOCA-1. To assess the level of agreement between the cross-correlation analysis and the GCA, we grouped filopodia into subclusters according to the Granger causality definition, which also produced a TOCA-1-responding subpopulation with greater mean cross-correlation between tip fluorescence and tip movement (Fig. 2G). To test whether the level of cross-correlation was significant (for either approach), we prepared 1000 simulated datasets by reshuffling the order of tip movement data for each filopodium. The mean CCF score for the TOCA-1-responding subcluster was significantly higher in the observed data than in the randomised data: none of the 1000 simulations produced a TOCA-1-responding subcluster with higher mean CCF score than that of the subcluster defined by cross-correlation analysis, and three of the 1000 simulations produced a subcluster with a higher CCF score than that of the subcluster defined by GCA (Fig. 2H). Thus, two independent statistical approaches validate the functional importance of the relationship between TOCA-1 and filopodial protrusion.

### The effect of mutations in TOCA-1 supports a functional role of TOCA-1 in filopodial protrusion

**Fig. 3.**
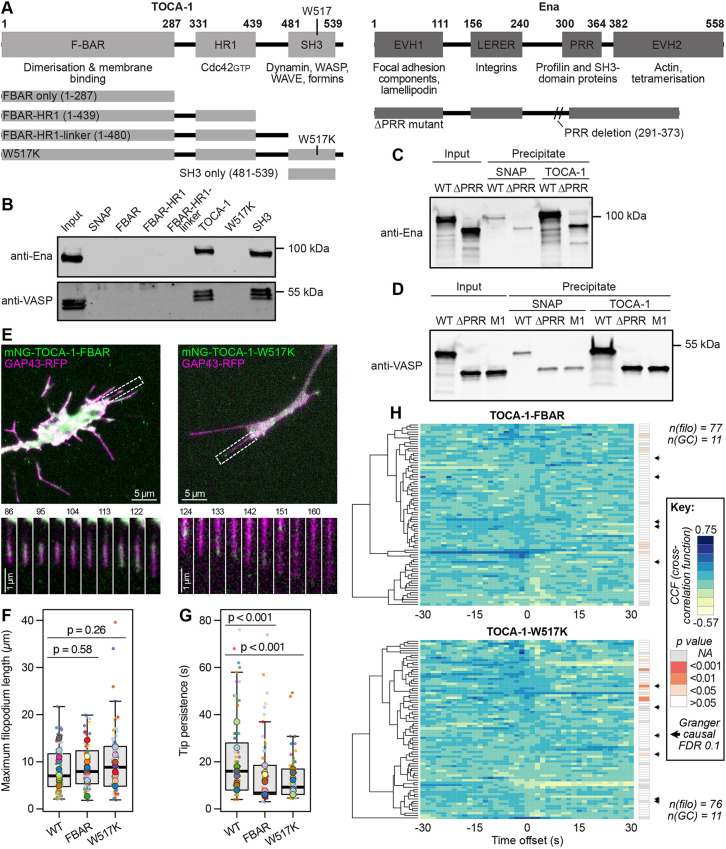
**An intact TOCA-1 SH3 domain is necessary for TOCA-1 function in filopodia.** (A) Schematic of TOCA-1 and Ena domain structures and mutations used in this study, numbered according to *Xenopus* amino acid sequences. Domain functions and binding partners are indicated. (B) SNAP-tagged full-length and mutant forms of TOCA-1, or SNAP alone, were coupled to beads and incubated with HSS. Western blotting of proteins that precipitated with the beads revealed that the intact TOCA-1 SH3 domain is necessary and sufficient to bind Ena and VASP in HSS. (C) Purified TOCA-1 and Ena interact directly via the PRR of Ena, as indicated by bead precipitation assays using SNAP-tagged TOCA-1 or SNAP alone with either wild-type (WT) or ΔPRR forms of Ena. (D) Purified VASP and TOCA-1 interaction is not abolished by removal of the PRR or additional proline removal (M1, ΔPRR with mutation of an additional proline), as indicated by bead precipitation assays using SNAP-tagged TOCA-1 or SNAP alone with WT, ΔPRR and M1 forms of VASP. (E) Imaging of mNG-tagged TOCA-1 mutants in RGCs reveals that truncation of TOCA-1 to the F-BAR domain only or a W517K mutation in the SH3 domain (Movie 3) leads to fewer and more diffuse TOCA-1 puncta. Dashed boxes indicate regions shown in montages beneath the main images. Time is indicated in seconds. (F) The maximum length reached by filopodia on average was unchanged between growth cones expressing wild-type TOCA-1 and growth cones expressing the indicated TOCA-1 mutants. (G) The median tip persistence was reduced after expression of either TOCA-1 mutant. Box plots as in Fig. 1E. Circles show the median for each growth cone, with matched coloured dots for each filopodium. *n* values for WT as in Fig. 2C; *n* values for mutants as in H. Significance assessed with Mann–Whitney tests, with multiple comparisons corrected using the Holm method. (H) Repeating the cross-correlation analysis, as in Fig. 2C, for the indicated TOCA-1 mutants shows a greatly reduced correlation between tip fluorescence and tip movement in either mutant. Black arrows indicate filopodia where TOCA-1-FBAR or TOCA-1 W517K fluorescence was Granger causal for tip movement, which could be due to heterodimerisation with endogenous TOCA-1. *P*-value assessed by Markov chain simulation (uncorrected for multiple comparisons). *n*(filo), number of filopodia; *n*(GC), number of growth cones.

To test the identified role of TOCA-1 in filopodial protrusion and tip persistence, we investigated the importance of different domains of TOCA-1. We confirmed that the SH3 domain was the major site of interaction between TOCA-1 and Ena or VASP by expressing each of the following TOCA-1 constructs, coupling them to magnetic beads and testing their ability to precipitate Ena and VASP: the F-BAR domain only, the F-BAR and HR1 domains, the F-BAR and HR1 domains with the subsequent linker region, full-length TOCA-1 with a point mutation in the SH3 domain that abolishes the interaction between TOCA-1 and N-WASP (W517K) (Ho et al., 2004), or the SH3 domain alone (Fig. 3A). A functional SH3 domain was found to be necessary and sufficient for Ena and VASP interactions (Fig. 3B). To confirm that the interactions with TOCA-1 were direct and via the proline-rich regions (PRRs) of Ena and VASP, we incubated purified recombinant wild-type and PRR-deleted (ΔPRR) forms of *Xenopus* Ena and VASP (Fig. 3A) with immobilised, purified SNAP–TOCA-1. Purified wild-type Ena and VASP bound directly to SNAP–TOCA-1 (Fig. 3C,D), and deleting the PRR of Ena reduced binding (Fig. 3C); however, neither PRR deletion nor mutation of an additional proline within VASP meaningfully reduced binding (Fig. 3D). VASP does not precipitate with SNAP beads alone (Fig. 3D), suggesting either that there are other possible candidate prolines within VASP or that there is a different type of molecular interaction sequence within VASP responsible for its interaction with TOCA-1. We note, however, that VASP behaves differently to Ena, with VASP being more dependent on polymerised actin for recruitment to *in vitro* filopodia-like structures and showing a lower level of correlation with TOCA-1 (Dobramysl et al., 2021) as well as the dynamic differences in growth cones (Urbančič et al., 2017).

In RGCs expressing mNG-tagged TOCA-1 F-BAR domain (mNG–TOCA-1-FBAR) or TOCA-1 W517K point mutant (mNG–TOCA-1-W517K), the TOCA-1 mutant puncta were more diffuse and transient (Fig. 3E; Movie 3) and their filopodial localisation was slightly reduced in comparison to the puncta formed by mNG–TOCA-1. Across all filopodia at any given timepoint, 48% of filopodia had TOCA-1-FBAR present in the shaft or tip, and 52% had TOCA-1-W517K (mean of three frames, from 11 growth cones with 48 and 46 filopodia on average, respectively), compared to 64% of filopodia for wild-type TOCA-1. Expression of the TOCA-1 mutants did not affect the average number of new filopodia per cell per minute (2.0 for RGCs expressing wild-type TOCA-1, 2.3 for RGCs expressing TOCA-1-FBAR and 2.3 for RGCs expressing TOCA-1-W517K) or filopodial lengths (Fig. 3F). Upon expression of the TOCA-1 mutants, filopodial tip persistence was reduced significantly, from a median of 16 s for RGCs expressing wild-type TOCA-1 to 6.2 s for RGCs expressing TOCA-1-FBAR and 9.2 s for RGCs expressing TOCA-1-W517K, suggesting dominant-negative effects (Fig. 3G), whereas filopodia bases moved faster but spent more time stalled in RGCs expressing the TOCA-1 mutants (Fig. S3D).

To test whether the shifts in localisation pattern and filopodial dynamics were functionally important, we repeated the cross-correlation analysis and GCA on the mutants. For both mutant forms of TOCA-1, the correlation between tip fluorescence and tip movement was almost completely lost (Fig. 3H), and fewer filopodia showed significant Granger causality between either mutant form of TOCA-1 and tip protrusion (black arrows in Fig. 3H), confirming that the mutations prevented TOCA-1 function, even though there was some remaining localisation to filopodia tips. Taken together, these results show that TOCA-1 function in filopodia is specific and dependent on a functional SH3 domain.

### Cdc42 inhibition with CASIN reduces levels of TOCA-1 and Ena at filopodia tips

To test whether TOCA-1 is specifically recruited to actively protruding filopodia and contributes to filopodial protrusion downstream of Cdc42, we treated RGCs with CASIN, a small-molecule inhibitor of Cdc42 that acts rapidly and reversibly at concentrations up to 20 µM but does not bind to other Rho GTPases (Peterson et al., 2006; Florian et al., 2012; Liu et al., 2019). An inactive CASIN analogue was found to have no effect on Cdc42, confirming the specificity of the effect. CASIN suppresses adhesion and migration of wild-type haematopoietic progenitor cells as strongly as a Cdc42 knockout, and CASIN treatment of Cdc42 knockout cells has no additional effect, demonstrating the specificity of this reagent (Liu et al., 2019).

**Fig. 4.**
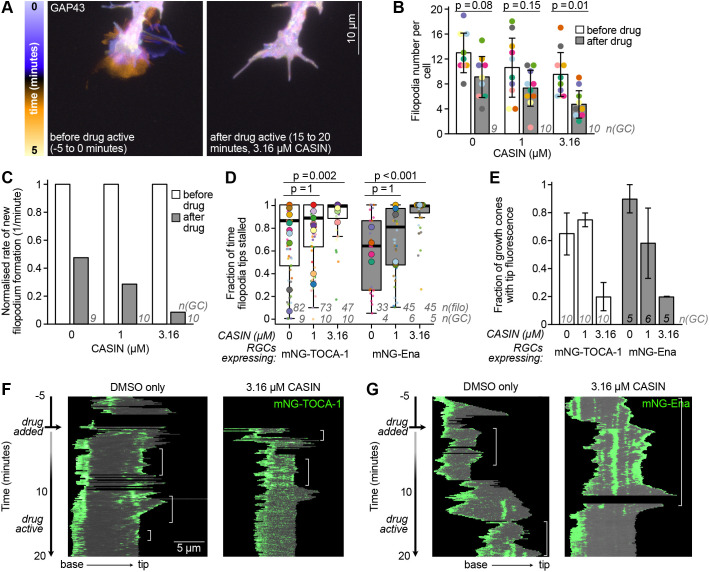
**Inhibition of Cdc42 with CASIN implicates TOCA-1 and Ena in Cdc42-driven filopodial protrusion.** (A) A temporal projection of a RGC expressing GAP43–RFP and mNG–TOCA-1 imaged every 7.5 s before and after treatment with Cdc42 inhibitor CASIN (3.16 µM), showing the loss of growth cone activity (see Movie 4). (B) The number of filopodia per growth cone before and after CASIN treatment, showing a moderate decrease at higher CASIN concentration. Circles show the mean for each growth cone, bars show the overall mean, error bars show the s.d. (C) The rate of new filopodia formation per minute before and after CASIN treatment, showing a large decrease in filopodial initiation upon CASIN treatment. Values are normalised to the rate before treatment, excluding filopodia that were longer than 2 µm in the first frame. Data in B and C are for RGCs expressing mNG–TOCA-1; results for mNG–Ena were similar (data not shown). (D) CASIN causes stalling of filopodia tips in RGCs expressing either mNG–TOCA-1 or mNG–Ena. Box plots as in Fig. 3F. (E) The fraction of growth cones that still had any filopodia tip fluorescence after CASIN treatment decreased in a dose-responsive manner for RGCs expressing mNG–TOCA-1 or mNG–Ena. Mean of two independent assessments, error bars show the range. Significance assessed in B and D using Mann–Whitney tests, with multiple comparisons corrected using the Holm method. *n*(filo), number of filopodia; *n*(GC), number of growth cones. (F) Representative example of filopodium growth and retraction with mNG–TOCA-1 fluorescence over the time course of CASIN or DMSO addition shown in a kymograph with filopodium base on the left and tip on the right. Time periods with tip fluorescence are indicated by white brackets. In the presence of CASIN, TOCA-1 fluorescence disappears, one round of protrusion continues, and then both TOCA-1 localisation and filopodial protrusion cease. (G) Individual representative kymographs, as in F, showing mNG–Ena fluorescence similarly disappearing during the last round of filopodial protrusion following addition of CASIN.

We reasoned that acute inhibition of Cdc42 should interfere with filopodial protrusion, and that if TOCA-1 is involved in filopodial protrusion via its HR1 domain interaction with Cdc42, TOCA-1 localisation would be disrupted. Time-lapse videos of growth cones showed that acute inhibition of Cdc42 led to arrest of filopodial and lamellipodial dynamics (Fig. 4A; Movie 4), with only a mild reduction in filopodia numbers (Fig. 4B). CASIN treatment strongly limited initiation of new filopodia in a dose-responsive manner (Fig. 4C) and led to almost complete stalling of existing filopodia tips after 20 min of treatment (Fig. 4D). Loss of TOCA-1 at filopodia tips was associated with cessation of tip protrusion (Fig. 4E–G). Most growth cones still had tip fluorescence after 20 min of DMSO treatment (Fig. 4E), but after treatment with a high concentration of CASIN, this dropped sharply for both mNG–TOCA-1- and mNG–Ena-expressing growth cones. After CASIN treatment, TOCA-1 was present up to the start of the last protrusion cycle, but no further cycles of protrusion were possible after loss of TOCA-1, whereas TOCA-1 puncta were often still present when filopodia tips stalled after DMSO control treatment (Fig. 4F). Growth cones expressing mNG–Ena showed a similar pattern (Fig. 4G). This confirms that both TOCA-1 and Ena are specifically recruited to protruding filopodia and respond to Cdc42-related changes in filopodial activity.

### A transient interaction of TOCA-1 and Ena in filopodial initiation

**Fig. 5.**
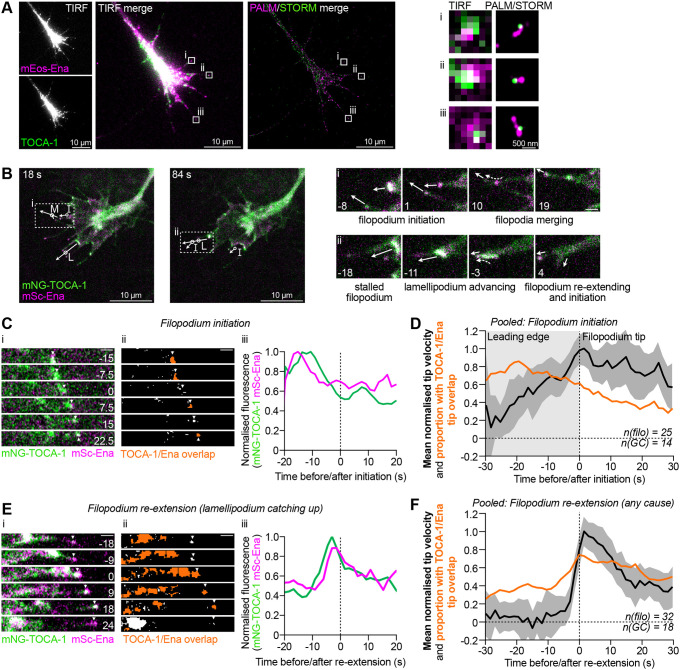
**TOCA-1 and Ena coincide transiently during filopodial initiation and re-extension.** (A) Two-colour PALM–STORM imaging of fixed RGCs expressing mEos–Ena (magenta) and immunostained to detect TOCA-1 (green). TIRF reference images are shown on the left. PALM–STORM imaging confirms that many filopodia have both Ena and TOCA-1 at their tips (see features i–iii, indicated by boxes and shown in enlarged images on the right); however, there is no consistent arrangement of the two proteins, indicating that there is no stable, structured complex. Images shown are representative of five cells. (B) In total, 57 filopodial protrusion events were collected from two-colour, simultaneous TIRF imaging (3 min at 1.5 s per frame) of RGCs injected with mNG–TOCA-1 and mScarlet–Ena (mSc–Ena), and assigned as initiation (25 of 57) or re-extension (32 of 57) events. In these examples (both from Movie 5), multiple protrusion events were observed, and some filopodia were observed undergoing two types of events. Arrows indicate ongoing or future tip movement for selected protruding filopodia, with circles showing site of protrusion events (I, initiation; L, re-extension after lamellipodium catching up; M, re-extension after merging of a second filopodium). Regions indicated by boxes i and ii are presented as expanded montages (right) to show the time course of compound protrusion events. Arrows indicate ongoing or future tip movement, dashed arrows indicate movement of fluorescent TOCA-1 and Ena puncta towards tips. (C) Montages of filopodium initiation, imaged as described in B, showing (i) the advancing tip position (arrows) and (ii) the presence of overlap between TOCA-1 and Ena (orange regions in thresholded images), and (iii) the fluorescence intensity of TOCA-1 and Ena before and after filopodium initiation. (D) Pooled data from 25 initiation events confirm that the prevalence of overlapping TOCA-1 and Ena at tips (orange line) was high before initiation before gradually falling to low levels, while the mean velocity of the tip peaked around initiation (black line, shading represents 95% c.i.). (E) Montages of a re-extension event caused by the lamellipodium catching up with a static filopodium, imaged as described in B, showing (i) the advancing tip position (arrows) and (ii) areas of overlapping TOCA-1 and Ena (orange regions in thresholded images), including large areas of the lamellipodium, and (iii) the fluorescence intensity of TOCA-1 and Ena at the tip, showing a peak shortly before re-extension. (F) Pooled data from 32 re-extension events confirms that levels of overlapping TOCA-1 and Ena (orange line) at static tips are low, rising to high levels around re-extension, as seen by the transient burst of high mean tip velocity (black line, shading represents 95% c.i.). All scale bars 1 µm unless indicated otherwise. Time indicated in seconds relative to initiation or re-extension events. *n*(filo), number of filopodia; *n*(GC), number of growth cones. See Fig. S4 for single-channel images of B, C and E.

TOCA-1 and Ena display a clustering-dependent interaction *in vitro*, display similar dynamics in filopodia and response to Cdc42 inhibition, and partly overlap in filopodia. Two-colour single-molecule localisation microscopy offers an opportunity to examine protein-specific ultrastructure and colocalisation at ∼50 nm resolution, so we combined expression of mEos-tagged Ena (mEos–Ena) and photoactivated localisation microscopy (PALM) with immunostaining of TOCA-1 and stochastic optical reconstruction microscopy (STORM) using Alexa Fluor 647-conjugated anti-rabbit IgG secondary antibodies. Our combined PALM–STORM approach resolved filopodia tips at high resolution, confirming that Ena and TOCA-1 are directly juxtaposed at a portion of filopodia tips (Fig. 5A, regions i–iii).

We reasoned that two-colour dynamic imaging would allow us to test whether the partial colocalisation of Ena and TOCA-1 seen in fixed cells corresponds to a transient event that is nonetheless predictive of filopodial extension. We expressed mNG–TOCA-1 with mScarlet-tagged Ena (mScarlet–Ena) in RGCs and developed a manual analysis pipeline (that did not require filopodia boundaries to be marked in the second channel) to study whether TOCA-1 and Ena coincide during filopodial initiation and extension. TOCA-1 and Ena frequently colocalised during filopodial initiation, and when filopodia resumed extension after stalling (Fig. 5B; Movie 5; see Fig. S4 for single-channel images). At any given timepoint, 46% of filopodia had both TOCA-1 and Ena (mean of three frames, from 15 growth cones with 72 filopodia on average, with TOCA-1 and Ena each present in 60% and 63% of filopodia, respectively). During the videos, 57 of 202 filopodia underwent protrusion (initiation or re-extension) events. For each filopodium protrusion event, we tracked the filopodium tip and measured the intensity of mNG–TOCA-1 and mScarlet–Ena at the tip. We identified areas of overlap between TOCA-1 and Ena by setting an intensity threshold for each channel and selecting all areas with both proteins present with a minimum area of 0.06 μm^2^ (∼14 pixels, to avoid single pixel fluctuations) and maximum area of 1 μm^2^ (orange regions in panel ii in Fig. 5C,E). The presence or absence of overlap at the tip was plotted relative to tip velocity during filopodial initiation and re-extension (Fig. 5D,F).

During filopodial initiation (Fig. 5B, annotated I, and Fig. 5C), we tracked the movement and fluorescence at the predicted base (the region on the membrane nearest to where the filopodium will form) before filopodial initiation, then at the tip after initiation. Both TOCA-1 and Ena were abundant at the site of formation before protrusion, and typically both were present at the nascent filopodium tip, but after a few seconds one or both (especially TOCA-1) faded from the tip. To take one example, TOCA-1 and Ena were found to overlap before initiation (−15 s to 0 s), but the overlap was only sometimes present after initiation (Fig. 5C). This same pattern was observed when pooling multiple examples (Fig. 5D). Overlap of TOCA-1 and Ena at the predicted base was observed in ∼70% of initiating filopodia at all timepoints during the 30 s before initiation (Fig. 5D). After initiation, the proportion of filopodia tips with TOCA-1 and Ena overlap steadily fell to ∼30%, suggesting that the TOCA-1–Ena complex forms transiently at the plasma membrane around the moment of filopodial protrusion.

Filopodial re-extension events were most often preceded by an advancing lamellipodium catching up with a static filopodium tip (17 of 32 events; Fig. 5B, annotated L and shown in panel ii, and Fig. 5C), or otherwise often due to the merging of a second filopodium (11 of 32 cases), usually starting with the second tip contacting the first shaft (Fig. 5B, annotated M and shown in panel i). Frequently, one or both proteins (especially mNG–TOCA-1) were absent from the static tip before re-extension (Fig. 5E) and were provided by the joining lamellipodium or filopodium. For both TOCA-1 and Ena, peak fluorescence occurred just before re-extension, followed by a drop in tip fluorescence, especially for TOCA-1 (Fig. 5E, panel iii). Across 32 filopodia re-extension events, the resumption of filopodium tip protrusion coincided with a rapid increase in the proportion of filopodia tips with TOCA-1 and Ena overlap, from less than 40% of filopodia at 30 s to 8 s before re-extension, to ∼75% of filopodia at the moment of re-extension, steadily falling to 50% by ∼30 s after (Fig. 5F, orange line). The increase in filopodia with overlapping TOCA-1 and Ena precedes the increase in mean tip velocity (Fig. 5F, black line), suggesting that a transient TOCA-1–Ena complex is associated with restarting of filopodial extension at mature tips. Taken together, our observations support a model in which TOCA-1 and Ena transiently associate before and during filopodia initiation, and during re-extension.

## DISCUSSION

Imaging of actin regulators alongside lamellipodial and filopodial dynamics at high spatial and temporal resolution gives valuable information about how cell players that are involved at multiple cellular sites, with multiple roles, carry out each function. In this work, we have utilised quantitative analysis of fluctuations in protein recruitment to show that TOCA-1 localisation to filopodia has a functional role in filopodial protrusion, which we validated through statistical tests of causality and by mutagenesis and perturbation of TOCA-1 binding to Cdc42 and its interaction partners through the SH3 domain. We show that Ena is a functional interaction partner of TOCA-1, and we propose that TOCA-1 and Ena can form a regulatory complex at filopodia that transiently occurs before and during filopodium initiation and re-extension and stimulates protrusion. Our results complement and extend previous biochemical and perturbation studies which have shown that TOCA-1 and paralogues are involved in filopodial formation but have given a conflicting picture of whether TOCA-1 proteins promote or oppose protrusion formation (Ho et al., 2004; Bu et al., 2009; Hu et al., 2011; Saengsawang et al., 2012; Taylor et al., 2019). Our strategy of quantitative dynamic analysis allowed us to show that TOCA-1 causes filopodial protrusion and promotes tip persistence.

When there are complex and non-linear responses to perturbation of actin regulatory proteins (Skruber et al., 2020; Dimchev et al., 2021; Pokrant et al., 2023), multiple complementary approaches are needed with different strengths, such as careful acute or inducible perturbations (Ghosh et al., 2004; Koestler et al., 2013), which limit system adaptation though still move the system far from physiological levels of the target protein, and quantitative fluctuation analysis, which allows investigation in a near-native setting. Although our experiments were conducted by exogenous expression of TOCA-1, we showed that there was no significant disruption of filopodial dynamics due to the mNG tag or overexpression under these conditions.

### A role for TOCA-1 independent of membrane curvature sensing

TOCA-1 and other F-BAR domain proteins have well-described roles in endocytosis and promotion of positively curved membrane structures such as cytoplasmic tubular networks (Frost et al., 2008; Taylor et al., 2019; Ledoux et al., 2023), but there are precedents for these and related BAR domain proteins acting in negatively curved membrane structures (Qualmann and Kelly, 2000; She et al., 2002; Chitu et al., 2005; Guerrier et al., 2009; Shimada et al., 2010; Becalska et al., 2013; Zhai et al., 2022). This suggests that any membrane curvature preference of F-BAR domain proteins does not limit them to certain cellular functions. This could be possible because of alternative binding modes that target flat membrane (Frost et al., 2008; McDonald et al., 2015) or the presence of complex membrane curvatures in filopodia, such as those that have been observed in electron microscopy studies of dendritic filopodial precursors (Galic et al., 2014) and described theoretically (Mancinelli et al., 2021); however, we could not detect such complex curvatures in our single-molecule localisation microscopy images. Membrane fluctuations, producing both positive and negative curvatures, are proposed to recruit diverse BAR superfamily proteins with either curvature preference (Mattila et al., 2007; Mancinelli et al., 2021).

### TOCA-1 and other membrane adaptor proteins in filopodial formation

Whereas colocalisation of CIP4 with Mena at actin ribs in cortical neurons has been observed previously, our analysis of filopodia provides correlative and quantitative insight into the influence of TOCA-1 on filopodia and its cooperation with Ena in Cdc42-driven rather than Rac1-driven events (Hu et al., 2011; Saengsawang et al., 2012, 2013). As well as F-BAR domain protein paralogues, lamellipodin (Krause et al., 2004), IRSp53 (Disanza et al., 2013), and recently formin FMNL2 (Fox et al., 2022) have been shown to play comparable membrane adaptor roles to TOCA-1 by scaffolding Ena, VASP or other actin regulators. The variety of these different contributors might reflect the different filopodia being studied, alternative mechanistic pathways, signalling-dependent use of adaptors or redundancy.

IRSp53 appears to localise to filopodia tips and shafts (Nakagawa et al., 2003; Sudhaharan et al., 2019; Cheng and Mullins, 2020; Tsai et al., 2022). In contrast, TOCA-1 localises to discrete puncta, especially at filopodia tips. The multiple shaft puncta observed following immunostaining of endogenous TOCA-1 might correspond to the dynamic puncta observed moving up and down shafts in videos of mNG–TOCA-1, or to a pool of TOCA-1 not labelled by mNG–TOCA-1. Live imaging shows that puncta of TOCA-1 mostly localise to tips transiently during initiation and re-extension events, suggesting a specific role that is distinct from the more structural role of I-BAR proteins in stabilising curved filopodial membrane.

Similar to clusters of lamellipodin (Cheng and Mullins, 2020), TOCA-1 is first recruited to the plasma membrane, then moves laterally and coalesces into larger puncta that recruit Ena and sometimes develop into filopodia. Furthermore, the frequent observation of TOCA-1 puncta moving inwardly, often associated with filopodial formation, is consistent with size-dependent splitting of the TOCA-1 cluster to maintain appropriate stoichiometry, as has been observed for the lamellipodin–VASP complex (Cheng and Mullins, 2020), although other explanations are possible, such as coincident retrograde movement of a membrane vesicle (Nozumi et al., 2017; Gallop, 2020). Imaging of fixed cells did not capture a consistent arrangement of TOCA-1 and Ena, even at super-resolution; however, rapid, time-resolved super-resolution imaging could be useful in revealing any arrangement of the transient TOCA-1–Ena complex during filopodial protrusion and how it links to the membrane.

### The mechanisms by which TOCA-1 promotes filopodial protrusion

Although TOCA-1 and Ena interact directly *in vitro* they did not persistently colocalise in cells, suggesting that specific conditions are needed for their interaction, and we provide evidence that presence of a sufficient density of TOCA-1 is a key factor. The proportion of filopodia tips with TOCA-1 and Ena overlap was substantially higher before and during filopodium initiation and re-extension compared to that of static tips. Measuring the degree of overlap is a simplification to capture and quantify the coincidence of the two proteins, and quantifying the fluorescence intensity of each protein is consistent with a transient peak in abundance associated with dynamic filopodia events. Use of split GFP might prove helpful in future studies to monitor the direct interaction in cells.

As well as filopodial extension, TOCA-1 appears to have a particular role in filopodial initiation, since it arrives before Ena at sites of filopodial initiation, and overlap between TOCA-1 and Ena is more abundant at the tips of short (potentially young) filopodia. N-WASP, an activator of Arp2/3 complex and branched actin, binds TOCA-1 at both high and low densities. It is possible that when TOCA-1 is initially recruited to leading-edge membranes, at lower density, it promotes polymerisation of branched actin structures via N-WASP or WAVE, such as advancing lamellipodia (Ho et al., 2004; Fricke et al., 2009). Then, after attaining sufficiently high density by coalescence of multiple puncta, TOCA-1 might switch to promoting linear actin polymerisation via Ena and formins, leading to filopodial initiation or re-extension. The clustering of TOCA-1 required for Ena binding is similar to observations with VASP clustered on beads (Breitsprecher et al., 2008), and the increased processivity is similar to the activity of Ena on filaments clustered by fascin (Harker et al., 2019). TOCA-1-responding filopodia had strongly increased persistence of tip movement, suggesting that TOCA-1 promotes filopodia that are resistant to interruptions in protrusion, perhaps by contributing to the assembly of a robust complex of actin regulators, including Ena, at the filopodia tips.

Our results reiterate previous findings that TOCA-1 mutants lacking an intact SH3 domain lose their function in neuronal cells (Bu et al., 2009; Saengsawang et al., 2012), whereas the recruitment of TOCA-1 to EGF-stimulated filopodia in epithelioid cells is not reliant on the SH3 domain (Hu et al., 2011). In our assays, the TOCA-1 mutants still had some filopodial localisation, with cross-correlation analysis and GCA confirming their loss of function, demonstrating the power of these approaches. The remaining localisation was likely due to heterodimerisation with endogenous TOCA-1, as the mutants also caused a dominant-negative effect, thus supporting a role for clustered TOCA-1 in filopodial growth.

Further work is needed to explore the possible interplay between the TOCA-1–N-WASP–WAVE and TOCA-1–Ena complexes, to confirm whether they are spatially and temporally separated, and to dissect the relationship between advancing lamellipodia and protruding filopodia. Quantitative image analysis approaches build on the candidate list of filopodial regulators characterised by biochemical, genetic and chemical perturbation studies. They offer the opportunity to untangle these processes and compare the multiple roles for shared actin regulators at different cellular sites *in vivo* without widespread alterations in the regulatory balance of the actin architecture.

## MATERIALS AND METHODS

### Plasmids

pET-His-SNAP-TOCA-1 (*X. tropicalis*; GenBank BC080964), pCS2-His-SNAP-Ena (*Xenopus laevis*; GenBank BC073107), pET-KCK-VASP (*X. laevis*; GenBank BC077932) and a plasmid expressing SNAP alone were generated previously (Dobramysl et al., 2021). mNeonGreen coding sequence was supplied by Allele Biotechnology and Pharmaceuticals (Shaner et al., 2013), and pCS2-mNG-Ena and pCS2-mNG alone were generated previously (Urbančič et al., 2017). New vectors were generated by PCR (Phusion-HF, NEB) into parent vectors digested with FseI/AscI unless otherwise stated. pCS2-mNG-TOCA-1 was generated by sub-cloning TOCA-1 into the digested pCS2-mNG vector, with oligonucleotide primers: 5′-GCATGGCCGGCCACCATGAGCTGGGGTACTG-3′ and 5′-GGCGCGCCTTAGATATAAGTTACTGC-3′. Ena was subcloned using primers: 5′-GCATGGCCGGCCACCATGAGTGAACAGAGCATC-3′ and 5′-GGCGCGCCCTATGCGCTGTTTG-3′ into pCS2 vectors generated with mEos3.2 (amplified from pmEos3.2-N1, Addgene 54525, deposited by Michael Davidson and Tao Xu; Zhang et al., 2012) or mScarlet (amplified from pLifeAct_mScarlet_N1, Addgene 85054, deposited by Dorus Gadella; Bindels et al., 2017). GAP43–RFP was a gift from the Holt laboratory, Department of Physiology, Development and Neuroscience, University of Cambridge, UK. A version of His–Ena without SNAP was generated using the above Ena primers. Ena-ΔPRR was generated by replacement of S291–G373 with a linker sequence GGGGSSGG, using In-Fusion cloning (Takara Bio) with primer pairs 5′-TCATCATCACGAATTCAGGCCGGCC-3′ and 5′-ACCTGAAGAACCACCTCCTCCCACTCTCCGTTCCCTTTCCCATTCC-3′, and 5′-GGTGGTTCTTCAGGTGGATCAGAAGAGAATCGTGCTTTATC-3′ and 5′-GGCCGCGGCGCCAATGCATTGGGCC-3′, into the parent vector digested with EcoRI and NotI. VASP-ΔPRR was generated by removal of S116–S192, using In-Fusion cloning with primer pairs 5′-ACAATTCCCCTCTAGAAATAATTTTG-3′ and 5′-CACCCCCACCAGTCTCCAGTGCATCCAAGG-3′, and 5′-AGACTGGTGGGGGTGGAGGAAGCTCAGGTGG-3′ and 5′-TATCATCGATAAGCTTTAATGCGGTAG-3′, into the parent vector digested with HindIII and XbaI. The additional mutation in construct M1 (P234G) was generated by PCR (Pwo Master; Roche) using primers 5′-CCTCCCCAGTTGGTGGAGTGGGTGCAAAGCCAGACATAAGTCG-3′ and 5′-CGACTTATGTCTGGCTTTGCACCCACTCCACCAACTGGGGAGG-3′. TOCA-1 mutants were generated as shown in Fig. 3A. For mutants starting at the N terminus, the forward primer was 5′-GCATGGCCGGCCACCATGAGCTGGGGTACTG-3′ and the reverse primers were 5′-GGCGCGCCTTAGCTGTAGTCTTCAAAGGGATAGTC-3′ (FBAR only), 5′-GGCGCGCCTTATTGTGCTACAAGATGGTTAGCTTC-3′ (FBAR-HR1) and 5′-GGCGCGCCTTAAGCTGGGAGAGGTTCATCATC-3′ (FBAR-HR-linker). The SH3-only mutant was generated using primers 5′-GCATGGCCGGCCACCATGGGACACTGCAAATCAC-3′ and 5′-GGCGCGCCTTATAGAGTGATATCTATGTAGGATGTGG-3′, and the W517K mutant was generated using primers 5′-GATAAAGGGGATGGAAAGACAAGAGCAAG-3′ and 5′-CTTGCTCTTGTCTTTCCATCCCCTTTATC-3′. Human TOCA-1 was sub-cloned into the pCS2-mNG vector using primers 5′-GATCGGCCGGCCATGAGCTGGGGCACGGAGC-3′ and 5′-GGCGCGCCCTGCAGCTCGAGTCAGGAACC-3′.

### Protein expression and purification

His-tagged proteins were expressed in BL21 pLysS *Escherichia coli* (Invitrogen) and purified with Ni-NTA columns (Qiagen) and gel filtration on S200 columns (Cytiva) as described previously (Dobramysl et al., 2021) with some exceptions. His–SNAP–TOCA-1 was purified in high salt buffers (300 mM NaCl instead of 150 mM NaCl during washing and elution), with two additional washes with 50 mM imidazole elution buffer before elution in a single 300 mM imidazole step and concentration using a spin concentrator (Amicon 10,000 MWCO, Millipore) before proceeding to gel filtration. TOCA-1 mutants were expressed and purified in the same way as wild-type TOCA-1. His–Ena-ΔPRR was expressed in 293F cells [R79007, Thermo Fisher Scientific; which were transfected with 293fectin (Thermo Fisher Scientific) and cultured according to the manufacturer's instructions] and purified in the same way as wild-type Ena (Dobramysl et al., 2021), except that 300 mM NaCl, not 150 mM, was used in wash and elution buffers. His–KCK–VASP mutants were expressed in BL21 *E. coli* in the same way as Ena mutants. His–SNAP alone was purified with elutions at 100 mM and 300 mM imidazole.

### Protein coupling to beads at different densities and precipitation from *Xenopus* egg HSS extracts

For each reaction, 20–40 μl SNAP-Capture beads (NEB, S9145S) were pre-equilibrated in 150 mM NaCl, 20 mM HEPES (pH 7.4) and 0.1% TWEEN-20, then 500 μl of SNAP-coupled protein preparation was added overnight, under rotation at 4°C in buffer containing 150 mM NaCl, 20 mM HEPES (pH 7.4), 0.1% TWEEN-20 and 1 mM dithiothreitol (DTT). Beads were washed five times in bead wash buffer (150 mM NaCl, 50 mM Tris, 1 mM DTT and 0.1% TWEEN-20). The capacity of benzylguanine sites on beads was determined empirically for each protein, with 500 μl of 12 μM SNAP–TOCA-1 or mutant proteins, or 500 μl of 24 μM SNAP alone per 40 μl of beads. For varying the density of TOCA-1 on beads, 500 μl of 2 μM SNAP–TOCA-1 was coupled to 10 μl of beads (determined to be maximum capacity), 50 μl of beads (intermediate density) or 100 μl of beads (low density), then 90 μl, 50 μl or 0 μl, respectively, of uncoupled beads were added to make a final volume of 100 μl beads for each condition, and a control sample with 100 μl uncoupled beads was also prepared. In a second stage of coupling, 500 μl of 80 μM SNAP alone was then added to bind the remaining benzylguanine sites on the beads. To precipitate proteins from *Xenopus* egg HSS extracts, coupled beads were incubated for 1 h at 4°C with 400 μl *Xenopus* HSS [prepared as previously described (Walrant et al., 2015) and diluted to 4.17 mg/ml in 50 mM Tris, 150 mM NaCl, 2 mM DTT and energy mix comprising 50 mM phosphocreatine, 20 mM Mg-ATP (adjusted to pH 7.0 with Tris-base) and 20 mM MgCl_2_]. Magnetic SNAP-Capture beads were removed from solution using a DynaMag particle concentrator (Invitrogen), washed three times with bead wash buffer. 4× Laemmli sample buffer (300 mM Tris 6.8, 8% SDS, 50% glycerol, 20% β-mercaptoethanol) was added to input samples, beads and depleted solutions to equivalent concentrations, and heated at 90°C. To precipitate purified Ena and VASP, 100 μl of 1 μM protein was incubated with 10 μl of coupled beads for 1 h at 4°C.

### Antibody affinity purification

Affi-Gel 15 beads (Bio-Rad, 1536051) were equilibrated in 300 mM NaCl, 20 mM Na-HEPES pH 7.4, 2 mM EDTA and 2 mM DTT, then incubated with His–SNAP–TOCA-1 or SNAP alone for 4 h at 4°C under rotation. Next, 1 M monoethanolamine, pH 8, was added to block (1 h), then the TOCA-1-coupled beads were washed on the Affi-Gel column with the following buffers: 500 mM NaCl and 20 mM Na-HEPES, glycine-HCl pH 2.5, triethylamine pH 11.5. The antibody was raised against purified *Xenopus tropicalis* SNAP-TOCA-1 in rabbit by Cambridge Research Biochemicals. Serum samples were stored at −80°C. The harvest bleed serum was passed through the column coupled to SNAP alone, then the flow-through was applied to the column coupled to His–SNAP–TOCA-1 (2 h, room temperature, under rotation). The column was washed with 20 ml of 400 mM NaCl and 30 mM Na-HEPES pH 7.7, and with 3 ml of 300 mM NaCl and 10 mM Tris-HCl pH 7.2 before elution. Elution with acid was carried out using 100 mM glycine pH 2.5 and 300 mM NaCl, and was neutralised with 1.5 M Tris-HCl pH 8.8, followed by elution with base using 100 mM triethylamine pH 11.5 and 300 mM NaCl, with subsequent neutralisation in 2 M Tris-HCl pH 6.5. Elution fractions were screened by absorbance at 280 nm and pooled, before exchanging buffer to 10 mM K-HEPES, 100 mM KCl, 1 mM MgCl_2_, 100 nM CaCl_2_, pH 7.4 overnight.

### Western blotting and antibodies

All blots are representative of three replicates, and input lanes represent 5% of HSS before incubation with beads, or, for Fig. 3C,D, 5% of Ena/VASP protein without bead incubation. Samples for western blotting were separated on 4–20% gradient polyacrylamide gels (Mini-PROTEAN TGX, Bio-Rad, 456-1096) and transferred to nitrocellulose membranes by wet transfer in 25 mM Tris, 192 mM glycine, 0.1% SDS and 20% methanol for 1 h at 0.38 A (Bio-Rad Mini Trans-Blot Cell apparatus) or dry transfer (programme 0, iBlot 2; Thermo Fisher Scientific). Membranes were blocked in Tris-buffered saline containing 5% milk powder and 0.1% TWEEN-20 (20–60 min, room temperature) and stained with primary antibody in blocking solution (1 h at room temperature or 4°C overnight). Membranes were washed 3–5 times in Tris-buffered saline containing 0.5% milk powder and 0.1% TWEEN-20 for 5–10 min then incubated with 800CW-conjugated goat anti-rabbit IgG secondary antibody (LI-COR, 926-32211; 30–60 min, room temperature) before washing as before and imaging on a LI-COR BioSciences Odyssey Sa scanner. For testing density dependence, blots were quantified using LI-COR Unicorn software, with a Friedman test to assess differences across the unclustered, intermediate and clustered states.

Antibodies for blotting: affinity-purified anti-TOCA-1 antibody (described above) or other unpurified bleeds (1:500 dilution). The anti-Ena (1:15,000), anti-VASP (1:500) and anti-N-WASP (1:2000) primary antibodies were affinity purified and described previously (Dobramysl et al., 2021), as was the anti-Diaph3 antibody (1:1300), a gift from Marc Kirschner (Harvard Medical School, Boston, MA, USA; Ho et al., 2004). Antibodies for immunostaining cells: affinity-purified anti-TOCA-1 antibody (described above; 1:500 dilution). Secondary antibodies used for immunostaining: Alexa Fluor 488-conjugated goat anti-rabbit IgG (1:2000; Invitrogen, A11008), Alexa Fluor 647-conjugated goat anti-rabbit IgG (1:2000; Invitrogen, A21244). For images of uncropped blots see Fig. S5.

### RGC preparation and injection of RNA

This research was regulated under the Animals (Scientific Procedures) Act 1986 Amendment Regulations 2012 following ethical review by the University of Cambridge Animal Welfare and Ethical Review Body. *Xenopus* embryos were fertilised *in vitro*, RNA was introduced by electroporation at stages 26–28, and RGC explants were taken at stages 35–36 and cultured for 19–24 h in 60% L-15 (Sigma-Aldrich, L1518) in water on 35 mm glass-bottom dishes (MatTek P35G-1.5-14-C) coated with 10 μg/ml poly-L-lysine (Sigma, P8920) for 1 h and 10 μg/ml laminin (Sigma, L2020) for 5–10 min, as described previously (Falk et al., 2007; Leung and Holt, 2008; Urbančič et al., 2017). For experiments with CASIN treatment, mScarlet–Ena, mEos–Ena or for immunostaining, 75 pg of RNA was micro-injected into the neural-fated blastomeres of 4-cell embryos instead of electroporation. mNG–TOCA-1 and mScarlet–Ena were co-injected at a ratio of 1:2 to equalise the resultant fluorescence levels. Capped RNA was synthesised after linearisation with NotI using an SP6 mMessage mMachine kit (Invitrogen, AM1340) with elution into RNase-free water.

### Live imaging of RGCs

Live imaging of RGCs was conducted in 60% L-15 in water under HILO illumination on a custom-made TIRF setup described previously (Urbančič et al., 2017) with an iLas2 illuminator (Roper Scientific), an Optosplit beam splitter (Cairn Research) and a CMOS camera (Hamamatsu ORCA-Flash4.0). Imaging of TOCA-1 mutants and experiments with CASIN treatment were acquired on a similar setup modified with a Multisplit beam splitter (Cairn Research) and a Kinetix CMOS camera (Photometrics) used in 12-bit (sensitivity) mode. Images were acquired at a rate of 2 s per timepoint (mNG–TOCA-1 and GAP43–RFP videos), 7.5 s per timepoint (CASIN treatment videos) or else 1.5 s per timepoint, with a 100×1.49 NA oil immersion objective (pixel size 0.065 μm) at room temperature, controlled by MetaMorph software (Molecular Devices). Where drugs were added, DMSO or CASIN (S6875, Stratech Scientific) dissolved in DMSO were diluted 1:500 in 60% L-15 in water to make a 2× solution. After 5 min of imaging (before drug addition), acquisition was paused, and half of the ∼350 µl RGC medium was removed and replaced with the same volume of 2× drug solution for a final DMSO concentration of 0.1%. The process of addition typically took 1–2 min, before acquisition was resumed.

### Imaging of fixed RGCs and PALM–STORM imaging

RGCs were washed once in 60% L-15 in water then fixed in PBS containing 4% paraformaldehyde and 7.5% sucrose (0.5–1 h, room temperature) before being washed three times in PBS containing 0.002% Triton X-100. Cells were permeabilised in PBS containing 0.1% Triton X-100 (3 min, room temperature) then washed twice as before and blocked in PBS containing 5% goat serum and 0.002% Triton X-100 (overnight, 4°C). Primary antibody was diluted in blocking solution and added to cells (1 h, room temperature) with three washes in PBS containing 0.5% goat serum and 0.002% Triton X-100 (5 min each). Secondary antibody was diluted in blocking solution and added to cells (30 min, room temperature) with phalloidin–Alexa Fluor 568 (1:100 dilution; Invitrogen, A12380), included where indicated, before washing as before. Cells were imaged in wash buffer by TIRF (as for live imaging), or for Fig. S1C and TOCA-1 and GAP43-RFP images (Fig. 1D), imaged with a Photometrics Evolve Delta EM-CCD camera instead.

For PALM–STORM imaging, the wash buffer was replaced with 150 μl STORM buffer [enzyme mix comprising 50 μg/ml catalase (Sigma), 50 mM Tris-HCl (pH 7.5) and 0.5 mg/ml glucose oxidase (Sigma); 100 mg/ml D-glucose (Sigma) in double-distilled water (ddH_2_O); and 100 mM cysteamine hydrochloride (MEA; Sigma, M6500) in ddH_2_O], and dishes were sealed by lowering a coverslip (18×18 mm) onto the central well. Images were acquired on an N-STORM system controlled by NIS Elements AR version 4.50 (Nikon), with an Agilent laser bed (405 nm, 488 nm, 561 nm and 647 nm lasers), CPI Plan Apo 100×1.49 NA objective and an N-STORM QUAD filter (405/488/561/647), as well as an iXon Ultra 897 EM-CCD camera (Andor). PALM–STORM images were acquired sequentially, in TIRF mode, with around 10,000 frames (20 ms per frame) of PALM imaging [using the 405 nm laser at low power (1–10%) to sparsely photoconvert mEos and the 561 nm laser at high power for imaging] followed by 20,000–30,000 frames (20 ms per frame) of STORM imaging (using the 405 nm laser at low power to tune blinking rates and 1–2 kW/cm^2^ 647 nm illumination). TIRF reference images (Fig. 5A) were acquired using the same system.

### Image processing and analysis

Image processing was performed in FIJI (Schindelin et al., 2012) with custom macros for analysis and some processing macros developed by Steve Rothery at the FILM facility, Imperial College London, UK (www.imperial.ac.uk/medicine/facility-for-imaging-by-light-microscopy/software/fiji/). Images were processed by overlaying the two channels (when the Optosplit beam splitter was used) and de-noising with nd-safir (for Fig. 1G and Movie 2) (Boulanger et al., 2010) or a 50–70 pixel rolling ball background subtraction for all other images. Videos with CASIN treatment were registered using Fast4DReg (without time averaging) to remove stage drift (Laine et al., 2019; Pylvänäinen et al., 2023).

For quantifying the prevalence of TOCA-1 or Ena in images of fixed or live filopodia, all filopodia protruding from the growth cone (not the axon) of length ≥3 μm were counted and scored visually for the presence of TOCA-1 or Ena in the filopodia shafts or tips. For quantifying overlap between TOCA-1 and Ena in filopodia from immunostained RGCs, individual filopodia of length ≥3 μm were extracted using the rotated rectangle tool, then measured using a straight line from the tip (defined by GAP43–RFP where present, else by the furthest punctum of Ena or TOCA-1 in line with the shaft) to the base (defined as the start of a region of consistent, narrow width). Puncta were counted automatically, after manual thresholding to exclude most of the noise, as particles between 0.06 μm^2^ (corresponding to ∼14 pixels, or a box of sides 250 nm) and 1.00 μm^2^. Overlap puncta were identified using a binary addition of the two thresholded images, and again automatically selecting resultant overlap puncta with area 0.06–1.00 μm^2^. Tip-localised puncta were defined as being at least half within a 1 μm diameter circle anchored to the tip. Significance was assessed with a two-tailed unpaired Student's *t*-test, if the data were normally distributed according to a Jarque–Bera test, or a Kruskal–Wallis test if not.

Videos of mNG–TOCA-1 or mNG–Ena and GAP43–RFP-expressing RGCs were analysed with Filopodyan (Urbančič et al., 2017) (without de-noising), using thresholding parameters: RenyiEntropy; Fit tip; erosion-dilation (ED) iterations, 4; Laplacian of Gaussian (LoG) sigma, 2.6–3.6; or other parameters that best segmented each video. The filter settings used were: minimum start frame, 1; minimum frames, 3; minimum–maximum length, 1.8; minimum length change, 0.1; maximum mean waviness, 0.38. Tracks assigned to filopodia that merged, moved out of focus or were otherwise poorly annotated were manually excluded.

Data tables were then analysed using FilopodyanR, with fluorescence signal processed by background subtraction based on signal near the growth cone boundary, then normalisation to growth cone body fluorescence. For plotting base fluorescence, the normalised time series were detrended by removing a linear trend, then mean base fluorescence at the predicted base plotted with a moving average (window size of five). Tip movement data was processed by removal of outliers (top and bottom 0.5%) then smoothing with a moving average with window size of three, or for data from CASIN-treated samples, a window size of five and removal of top and bottom 1% outliers (Urbančič et al., 2017). CCF scores were calculated and plotted using the ‘FilopodyanR CCF.R’ script, with time series filtered by minimum 50 frames. Missing values were not removed during CCF calculation.

Kymographs of TOCA-1 and Ena fluorescence were generated using the Filopodyan ‘Process Profile Graphs’ option, plotting the segmented filopodium base, shaft and tip as a straight line for each frame. The camera offset was subtracted from the raw fluorescence values, then the fluorescence values were normalised by the median fluorescence intensity in the growth cone body, to account for varying expression levels in different cells. Low-contrast images of the kymograph were used to mark the filopodium outline.

Videos of RGCs treated with CASIN were scored visually, comparing before (−5 min to 0 min) and after (15–20 min) DMSO or drug treatment, for the presence of one or more filopodia with a sustained, clear punctum of tip fluorescence. Data show the mean of two independent assessments made using datasets with the treatment groups masked. Temporal projections were prepared using the Temporal-Color Code plugin in Fiji.

TOCA-1 puncta behaviour was quantified from videos of cells expressing mNG–TOCA-1 and GAP43–RFP after de-noising. For scoring of numbers of coalescing puncta, we used the maximum number of distinct puncta that were observed during the <14 s before filopodial initiation. For quantifying the fate of TOCA-1 puncta, a macro was used to track all puncta with minimum five frames, minimum 0.65 μm link distance and starting within 1 μm of the leading edge. Then, individual puncta were randomly selected from a list to then be manually assigned to a category.

Videos of mNG–TOCA-1 and mScarlet–Ena were processed manually, with background subtraction, then extraction of any filopodia that underwent initiation or re-extension events by using the rotated rectangle tool in Fiji to re-orientate the filopodium such that the long axis of the filopodium was aligned with the image window. Fluorescence intensity values were extracted along the length of the filopodium (averaging across a ∼20 pixel column for each pixel along the long axis) using a line profile tool (line/time macro; FILM facility, Imperial College London, UK). Overlap puncta were defined as areas of overlap 0.06–1.00 μm^2^ after thresholding and binary addition, as for images of fixed samples above, and were scored as positive if any pixels in the ∼20 pixel column were part of an overlap punctum. The tip was tracked manually using TrackMate (Tinevez et al., 2017), and a custom Excel worksheet was used to extract the fluorescence intensity values and presence or absence of any overlap between TOCA-1 and Ena at the filopodium tip (averaged over a five pixel window centred on the tip along the long axis of filopodium). The velocity was calculated as displacement along the long axis for each frame, and velocity and fluorescence intensity values, as well as the proportion with overlap of TOCA-1 and Ena, were smoothed by a moving average over three frames.

### Granger causality analysis

GCA was conducted on the same processed time series used for cross-correlation analysis. To begin the analysis, the stationarity of our time series was confirmed via the Augmented Dickey–Fuller test (Dickey and Fuller, 1979). Next, we selected the optimum lag for the Granger causality test by selecting the lag from one to five that minimised the Bayesian information criteria (Schwarz, 1978). We tested Granger causality using the MATLAB function gctest(). Raw *P*-values were corrected for multiple hypothesis testing using the Benjamini–Hochberg FDR procedure (Benjamini and Hochberg, 1995).

### Super-resolution image reconstruction

Image data stacks were converted from Nikon image files into TIF stacks using Fiji. Single-molecule blinking events were detected in unprocessed camera frames and fit with a two-dimensional (2D) Gaussian model as previously described (Li et al., 2018). Fit results were filtered based on number of photons (50–5000), localisation precision (0.5–50 nm), goodness of fit (log likelihood ratio<150) and point spread function width (sigma, 50–150 nm). Post-processing drift correction was applied using a redundant cross-collection algorithm as previously described (Wang et al., 2014). Because PALM and STORM datasets were collected sequentially, in that order, drift correction was applied relative to the last frame of the PALM image and to the first frame of the STORM image. Images were reconstructed with a 16 nm pixel size and blurred with a 2D Gaussian equal to the average localisation precision of the image.

## Supplementary Material

10.1242/joces.261057_sup1 Supplementary information

## First Person

This article has an associated First Person interview with the first author of the paper.
