# Supplementary material for: Filopodial protrusion driven by density-dependent Ena–TOCA-1 interactions
Source: J Cell Sci. 2024 Mar 21;137(6):jcs261057. doi: 10.1242/jcs.261057 (PMC11006392; doi:10.1242/jcs.261057)
Supplement: Supplementary information [file joces-137-261057-s1.pdf]

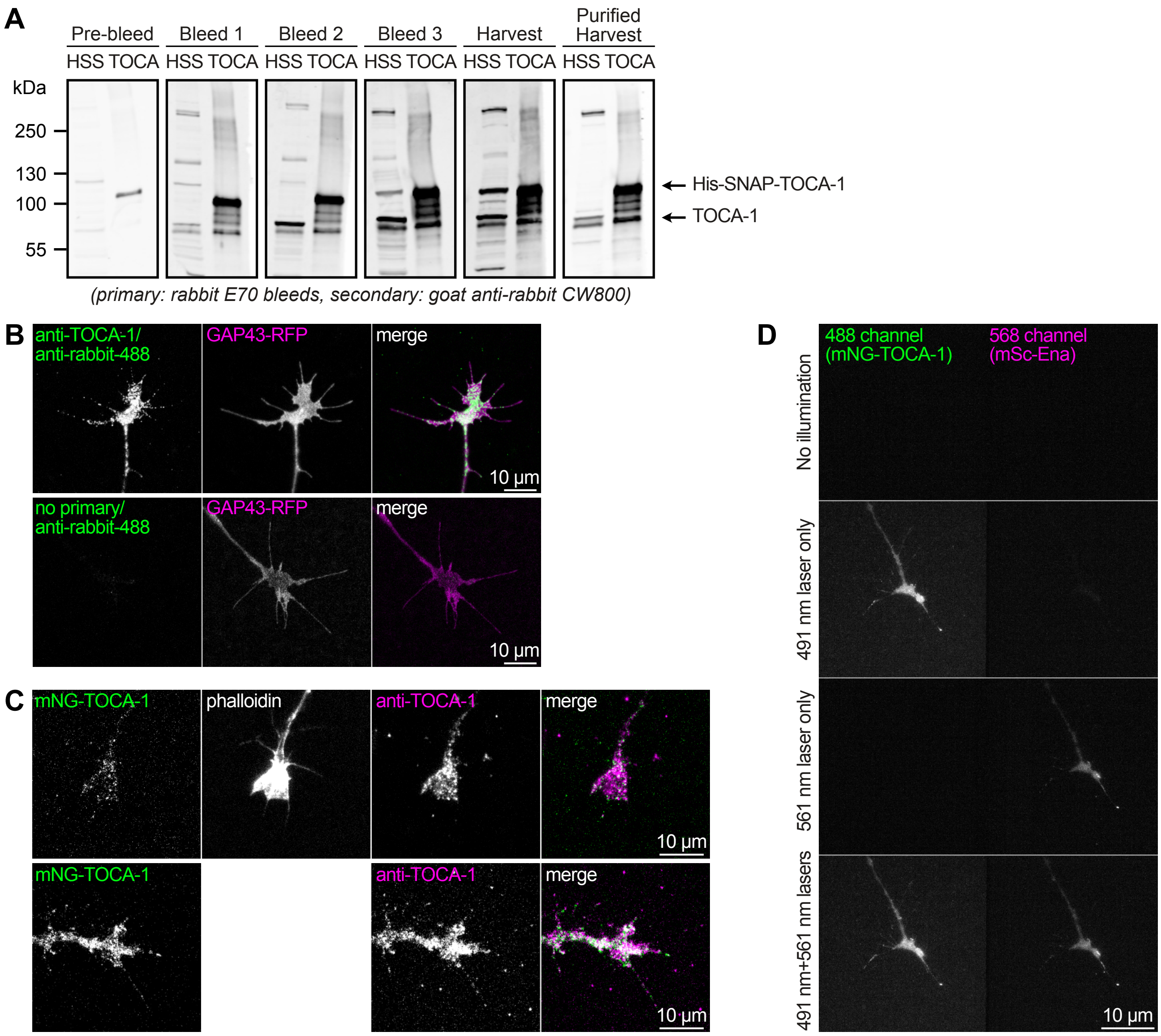

Fig. S1. Affinity purified rabbit anti-TOCA-1 antibody recognises TOCA-1 in lysate and cells.

(A) SDS-PAGE gel with *Xenopus* egg extract (HSS) or purified SNAP-TOCA-1 (TOCA), stained with the indicated bleeds at 1:500 dilution, showing that before immunisation, a small non-specific band was present with concentrated, purified SNAP-TOCA-1 and not with HSS. All post-immunisation bleeds recognise TOCA-1 in HSS and purified SNAP-TOCA-1, and after affinity purification ("purified harvest") the specificity is greatly improved. (B) RGCs expressing membrane marker GAP43-RFP. No primary antibody immunostaining control, showing that fluorescence is specific to anti-TOCA-1/anti-rabbit-488. Contrast applied equally between images for 488 channel and allowed to vary in GAP43-RFP channel to account for variable expression levels. (C) RGCs expressing mNG-TOCA-1, immunostained with anti-TOCA-1 / anti-rabbit-AF647 and phalloidin-AF568, showing a similar pattern of fluorescence between endogenous and exogenous TOCA-1. (D) Acquiring images with no, one or both lasers active confirmed that almost no fluorescent signal in the 568 channel was due to excitation of either fluorophore with the 491 nm laser, and vice versa. No background subtraction, contrast applied equally to all eight images.

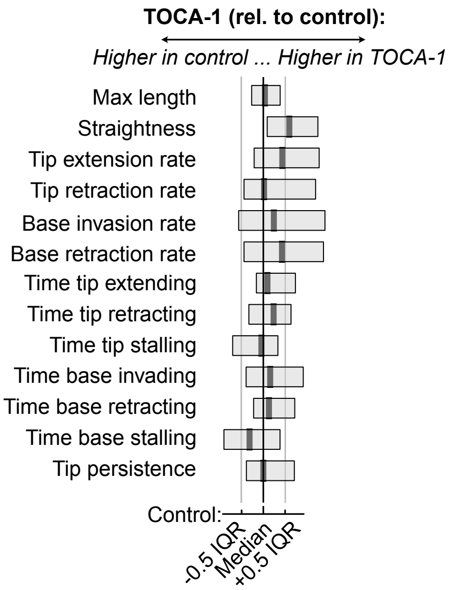

Fig. S2. Overexpression of mNG-TOCA-1 does not affect filopodial dynamics
Filopodia from RGCs electroporated with mRNA for membrane marker GAP43-RFP and either mNG-TOCA-1 or mNG alone were analysed with Filopodyan, revealing no significant differences in filopodia parameters. Boxes show median and IQR of mNG-TOCA-1 data, grid lines show median and IQR for control data; see Table S1 for full data.

Journal of Cell Science • Supplementary information

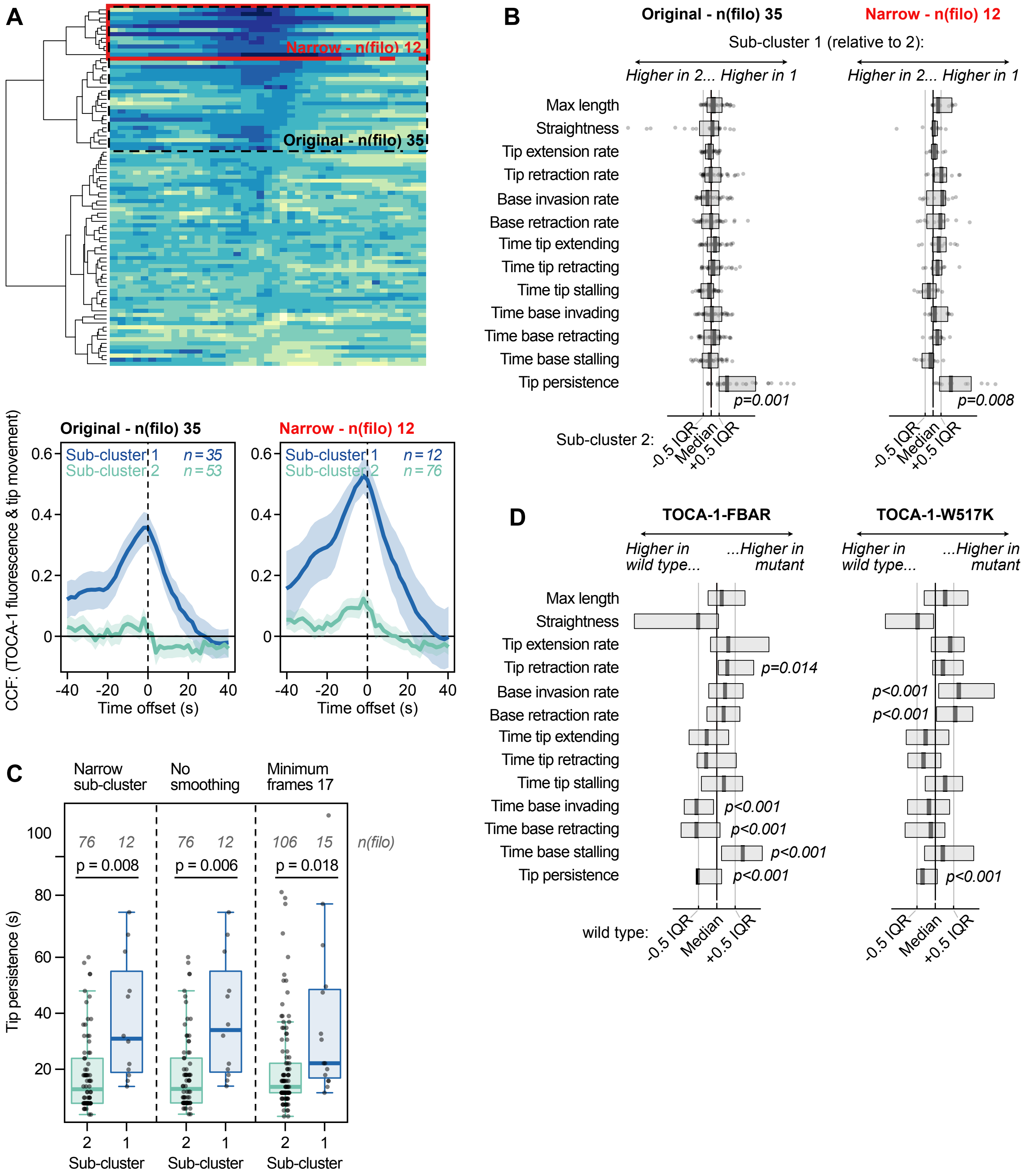

Journal of Cell Science • Supplementary information

Fig. S3. TOCA-1 tip fluorescence correlates with tip movement, producing a high tip persistence sub-cluster, across varying processing parameters
(A) Varying the sub-cluster point after hierarchical clustering (heatmap adapted from Fig. 2C) still produces a TOCA-1 responding sub-cluster (high mean cross-correlation score peaking with a lag of -2 s). "Original" line graph adapted from Fig. 2D. (B) Both the original and narrow sub-clusters have significantly higher median tip persistence, but no other significant morphological changes. (C) Varying data processing parameters such as altering the sub-clustering point (see (A)), removing smoothing of tip movement data or reducing the minimum frames threshold for inclusion of a filopodium still produces a TOCA-1 responding sub-cluster with significantly higher tip persistence than non responding filopodia. (D) Both TOCA-1 mutants led to significantly reduced tip persistence, with other significant changes including in filopodial base dynamics. Significance tested by Mann-Whitney test after Holm correction for multiple comparisons and shown where $p < 0.05$, n(filo) shown on graphs; all data n(GC) = 11.

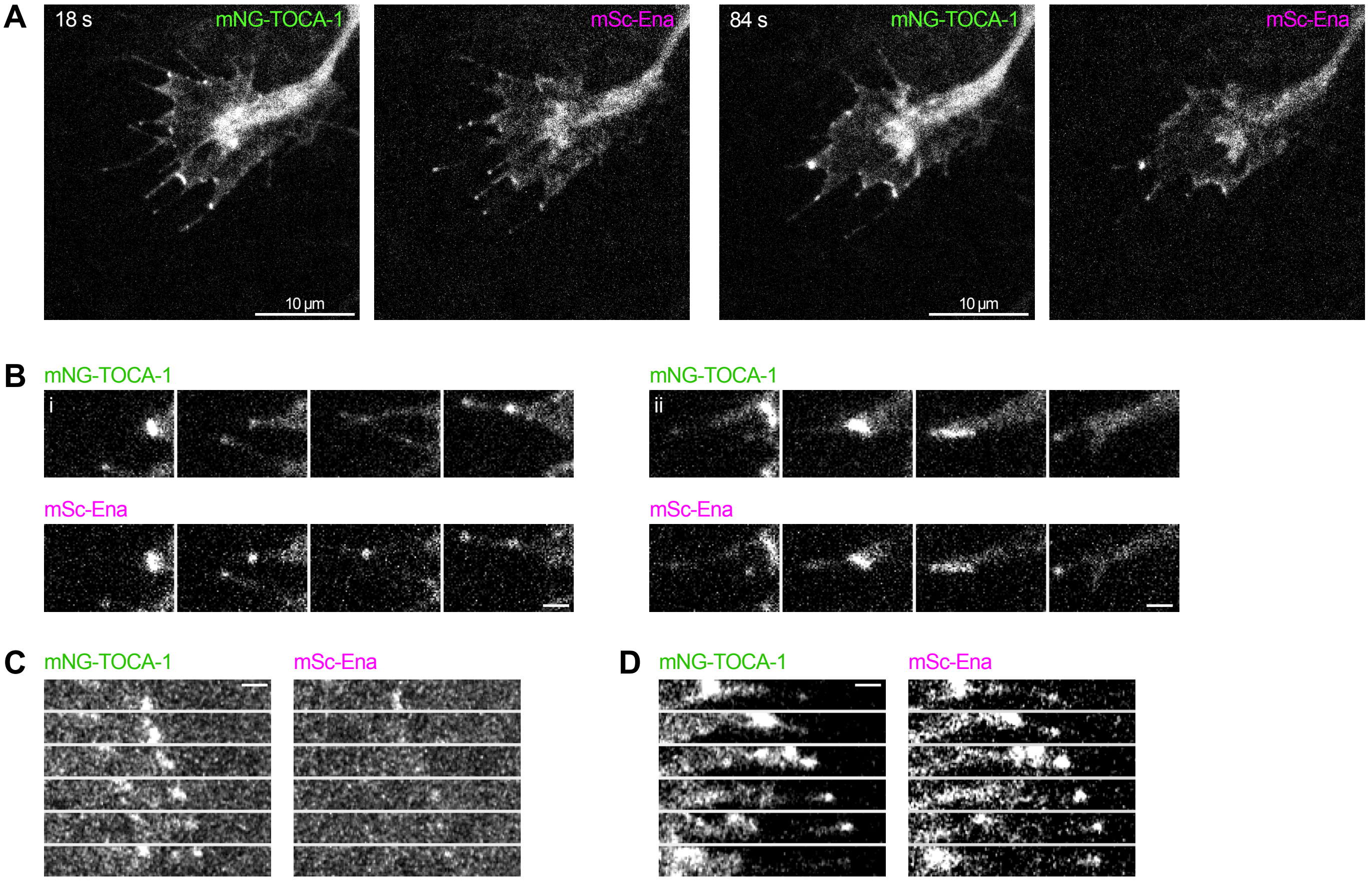

Fig. S4. Single channel images of RGCs expressing mNG-TOCA-1 and mSc-Ena
Images of mNG-TOCA-1 and mSc-Ena relating to (A) Fig. 5B, (B) Fig. 5Bi and Bii, (C) Fig. 5Ci, and (D) Fig. 5Ei. Scale bars 1 µm unless stated.

Journal of Cell Science • Supplementary information

J. Cell Sci.: doi:10.1242/jcs.261057: Supplementary information

Blots from Figure 1A

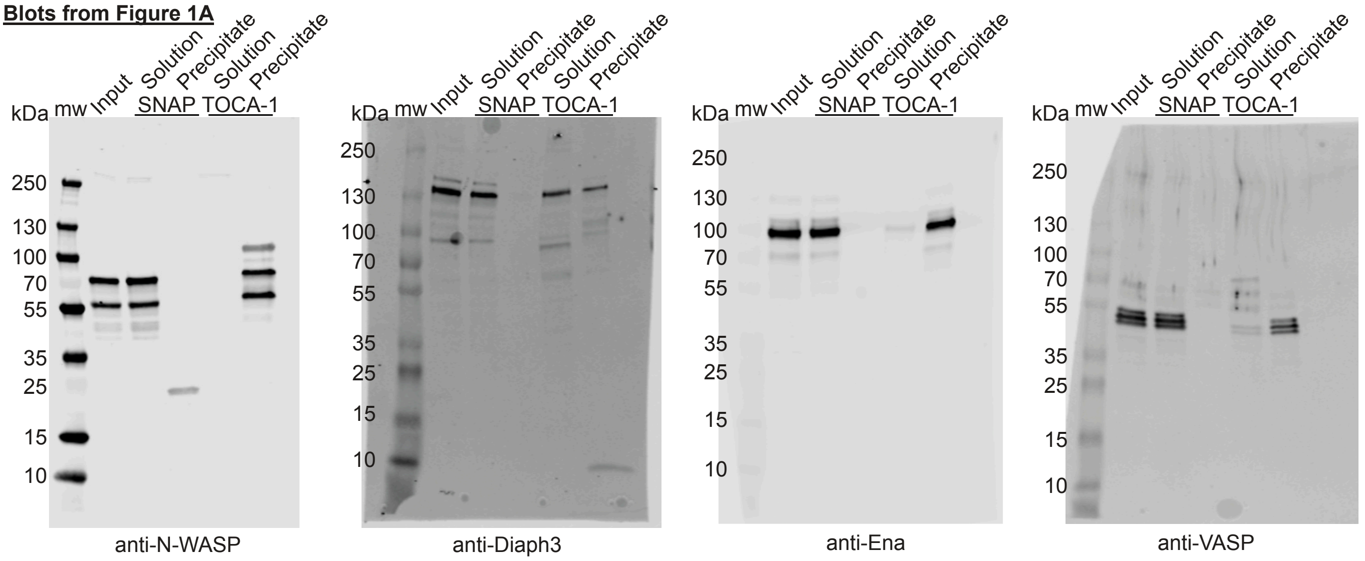

Blots from Figure 1B

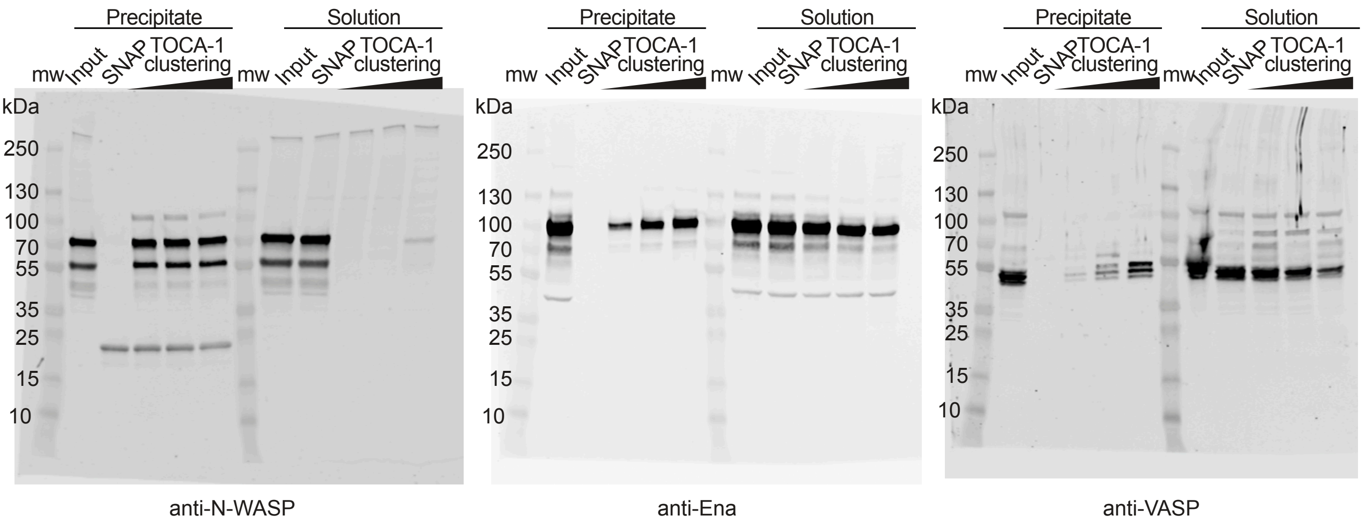

Blots from Figure 3B

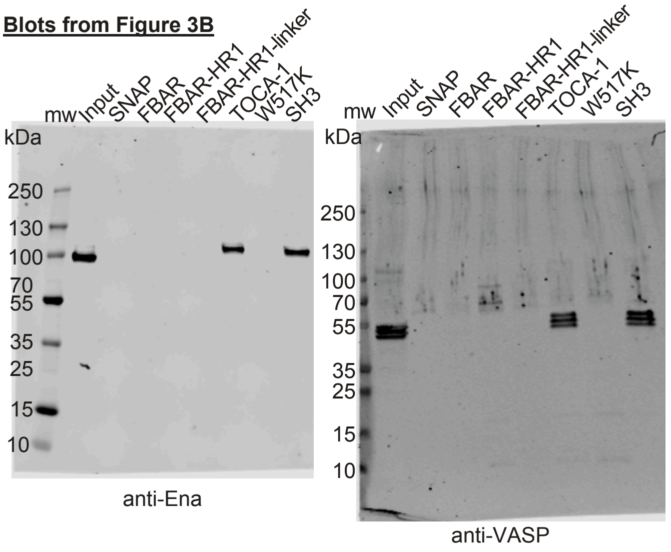

Blot from Figure 3C

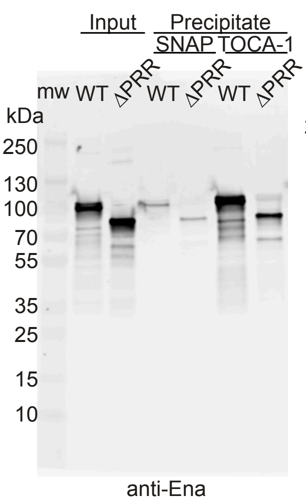

Blot from Figure 3D

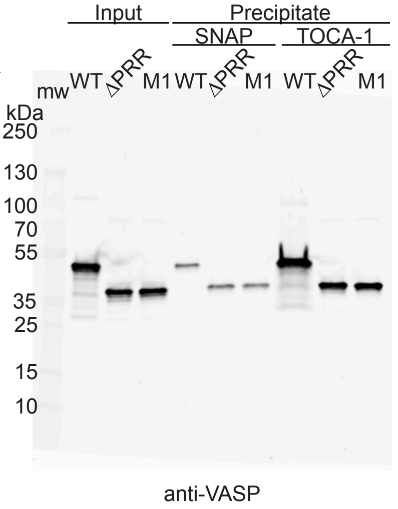

Fig. S5. Uncropped Western blots relating to Fig. 1 and Fig. 3

Journal of Cell Science • Supplementary information

Table S1. Filopodia dynamics parameters from Filopodyan, showing no difference between mNG alone and mNG-TOCA-1.

TOCA-1 vs CTRL (Fig S2)

Parameter	*Condition*	*Min.*	*1st Qu.*	*Median*	*Mean*	*3rd Qu.*	*Max.*	*SD*	*N*
Max length	CTRL	2.000	2.718	4.993	6.783	11.212	17.039	4.74	63
Max length	TOCA-1	1.852	2.798	5.292	6.536	8.162	21.708	4.63	80
Straightness	CTRL	0.652	0.882	0.897	0.883	0.918	0.935	0.06	31
Straightness	TOCA-1	0.607	0.900	0.918	0.892	0.941	0.980	0.09	41
Tip extension rate	CTRL	0.038	0.045	0.053	0.061	0.070	0.124	0.02	41
Tip extension rate	TOCA-1	0.035	0.048	0.064	0.073	0.084	0.181	0.03	62
Tip retraction rate	CTRL	0.033	0.051	0.055	0.060	0.070	0.112	0.02	35
Tip retraction rate	TOCA-1	0.035	0.047	0.055	0.070	0.078	0.198	0.04	50
Base invasion rate	CTRL	0.034	0.047	0.053	0.060	0.063	0.128	0.02	43
Base invasion rate	TOCA-1	0.035	0.045	0.057	0.061	0.075	0.128	0.02	66
Base retraction rate	CTRL	0.035	0.045	0.055	0.056	0.062	0.101	0.02	46
Base retraction rate	TOCA-1	0.035	0.048	0.062	0.065	0.078	0.143	0.02	65
Time tip extending	CTRL	0.000	0.004	0.182	0.255	0.457	0.800	0.24	55
Time tip extending	TOCA-1	0.000	0.112	0.223	0.321	0.504	1.000	0.28	72
Time tip retracting	CTRL	0.000	0.000	0.052	0.113	0.162	1.000	0.17	55
Time tip retracting	TOCA-1	0.000	0.000	0.089	0.110	0.151	1.000	0.15	72
Time tip stalling	CTRL	0.000	0.412	0.649	0.632	0.879	1.000	0.29	55
Time tip stalling	TOCA-1	0.000	0.332	0.629	0.569	0.800	1.000	0.29	72
Time base invading	CTRL	0.000	0.017	0.121	0.133	0.198	0.667	0.13	57
Time base invading	TOCA-1	0.000	0.052	0.148	0.188	0.281	1.000	0.19	73
Time base retracting	CTRL	0.000	0.029	0.162	0.214	0.250	1.000	0.26	57
Time base retracting	TOCA-1	0.000	0.115	0.190	0.227	0.316	1.000	0.19	73
Time base stalling	CTRL	0.000	0.525	0.681	0.653	0.851	1.000	0.27	57
Time base stalling	TOCA-1	0.000	0.397	0.582	0.585	0.802	1.000	0.25	73
Tip persistence	CTRL	4.000	10.000	16.000	19.321	26.000	60.000	12.25	53
Tip persistence	TOCA-1	4.000	10.000	16.000	22.306	27.000	106.00	19.49	72

TOCA-1 vs CTRL (Fig S2)

	Fold change (median)	*Fold change (mean)*	*z-score*	*Cliff's delta*	*P (Mann-Whitney)*	*P (Holm-adjusted)*
Max length	1.0599	0.9636	-0.0520	-0.0222	0.8215	1.0000
Straightness	1.0234	1.0106	0.1520	0.3375	0.0143	0.1858
Tip extension rate	1.1987	1.2010	0.5745	0.1853	0.1134	0.9069
Tip retraction rate	1.0059	1.1552	0.4851	0.0629	0.6265	1.0000
Base invasion rate	1.0693	1.0189	0.0515	0.0254	0.8258	1.0000
Base retraction rate	1.1299	1.1499	0.5362	0.2475	0.0270	0.3237
Time tip extending	1.2275	1.2581	0.2691	0.1424	0.1693	1.0000
Time tip retracting	1.7121	0.9727	-0.0179	0.0523	0.6097	1.0000
Time tip stalling	0.9692	0.9006	-0.2187	-0.1258	0.2263	1.0000
Time base invading	1.2275	1.4077	0.4100	0.1901	0.0633	0.6960
Time base retracting	1.1695	1.0617	0.0515	0.1702	0.0967	0.9065
Time base stalling	0.8542	0.8965	-0.2544	-0.1735	0.0907	0.9065
Tip persistence	1.0000	1.1545	0.2436	0.0128	0.9042	1.0000

Journal of Cell Science • Supplementary information

Table S2. Filopodia dynamics parameters from Filopodyan, showing that TOCA-1 responding filopodia had significantly increased tip persistence compared to others.

Sub-cluster 1 vs Sub-cluster 2 (Fig. 3C)

Parameter	*Sub-cluster*	*Min.*	*1st Qu.*	*Median*	*Mean*	*3rd Qu.*	*Max.*	*SD*	*N*
Max length	2	2.119	4.213	6.933	8.170	11.518	21.708	4.82	53
Max length	1	2.077	5.041	8.115	8.698	11.938	17.232	4.35	35
Straightness	2	0.453	0.879	0.917	0.857	0.923	0.973	0.14	36
Straightness	1	0.687	0.885	0.920	0.896	0.937	0.980	0.07	26
Tip extension rate	2	0.036	0.057	0.070	0.101	0.111	0.602	0.09	45
Tip extension rate	1	0.034	0.050	0.064	0.065	0.077	0.100	0.02	34
Tip retraction rate	2	0.038	0.047	0.066	0.084	0.088	0.257	0.06	43
Tip retraction rate	1	0.039	0.050	0.065	0.075	0.092	0.150	0.03	35
Base invasion rate	2	0.034	0.054	0.070	0.074	0.087	0.178	0.03	51
Base invasion rate	1	0.039	0.050	0.062	0.068	0.086	0.113	0.02	35
Base retraction rate	2	0.036	0.057	0.066	0.069	0.078	0.134	0.02	51
Base retraction rate	1	0.042	0.053	0.066	0.067	0.077	0.116	0.02	35
Time tip extending	2	0.000	0.085	0.211	0.241	0.371	0.744	0.20	53
Time tip extending	1	0.000	0.140	0.280	0.269	0.356	0.603	0.17	35
Time tip retracting	2	0.000	0.017	0.127	0.130	0.191	0.435	0.12	53
Time tip retracting	1	0.021	0.100	0.151	0.172	0.220	0.443	0.11	35
Time tip stalling	2	0.148	0.380	0.661	0.629	0.856	1.000	0.26	53
Time tip stalling	1	0.102	0.356	0.600	0.559	0.733	0.969	0.23	35
Time base invading	2	0.000	0.153	0.205	0.213	0.297	0.463	0.11	53
Time base invading	1	0.059	0.143	0.212	0.221	0.302	0.400	0.10	35
Time base retracting	2	0.000	0.167	0.263	0.249	0.345	0.473	0.12	53
Time base retracting	1	0.042	0.183	0.297	0.281	0.356	0.482	0.12	35
Time base stalling	2	0.159	0.390	0.517	0.538	0.667	1.000	0.21	53
Time base stalling	1	0.164	0.361	0.492	0.498	0.637	0.885	0.19	35
Tip persistence	2	4.000	8.000	10.000	16.038	18.000	60.000	12.89	53
Tip persistence	1	8.000	15.000	20.000	28.400	38.000	76.00	18.61	35

Sub-cluster 1 vs Sub-cluster 2 (Fig. 3C)

	Fold change (median)	*Fold change (mean)*	*Cliff's delta*	*P (Mann-Whitney)*	*P (Holm-adjusted)*
Max length	1.1705	1.0646	0.1008	0.4278	1.0000
Straightness	1.0039	1.0459	0.1325	0.3830	1.0000
Tip extension rate	0.9275	0.6456	-0.2170	0.1013	1.0000
Tip retraction rate	0.9798	0.8985	0.0545	0.6841	1.0000
Base invasion rate	0.8914	0.9270	-0.1126	0.3794	1.0000
Base retraction rate	0.9987	0.9650	-0.0370	0.7751	1.0000
Time tip extending	1.3284	1.1151	0.1429	0.2602	1.0000
Time tip retracting	1.1854	1.3212	0.2345	0.0641	0.7695
Time tip stalling	0.9077	0.8894	-0.1682	0.1849	1.0000
Time base invading	1.0317	1.0368	0.0237	0.8546	1.0000
Time base retracting	1.1290	1.1294	0.1369	0.2808	1.0000
Time base stalling	0.9508	0.9256	-0.0895	0.4818	1.0000
Tip persistence	2.0000	1.7708	0.4868	0.0001	0.0014

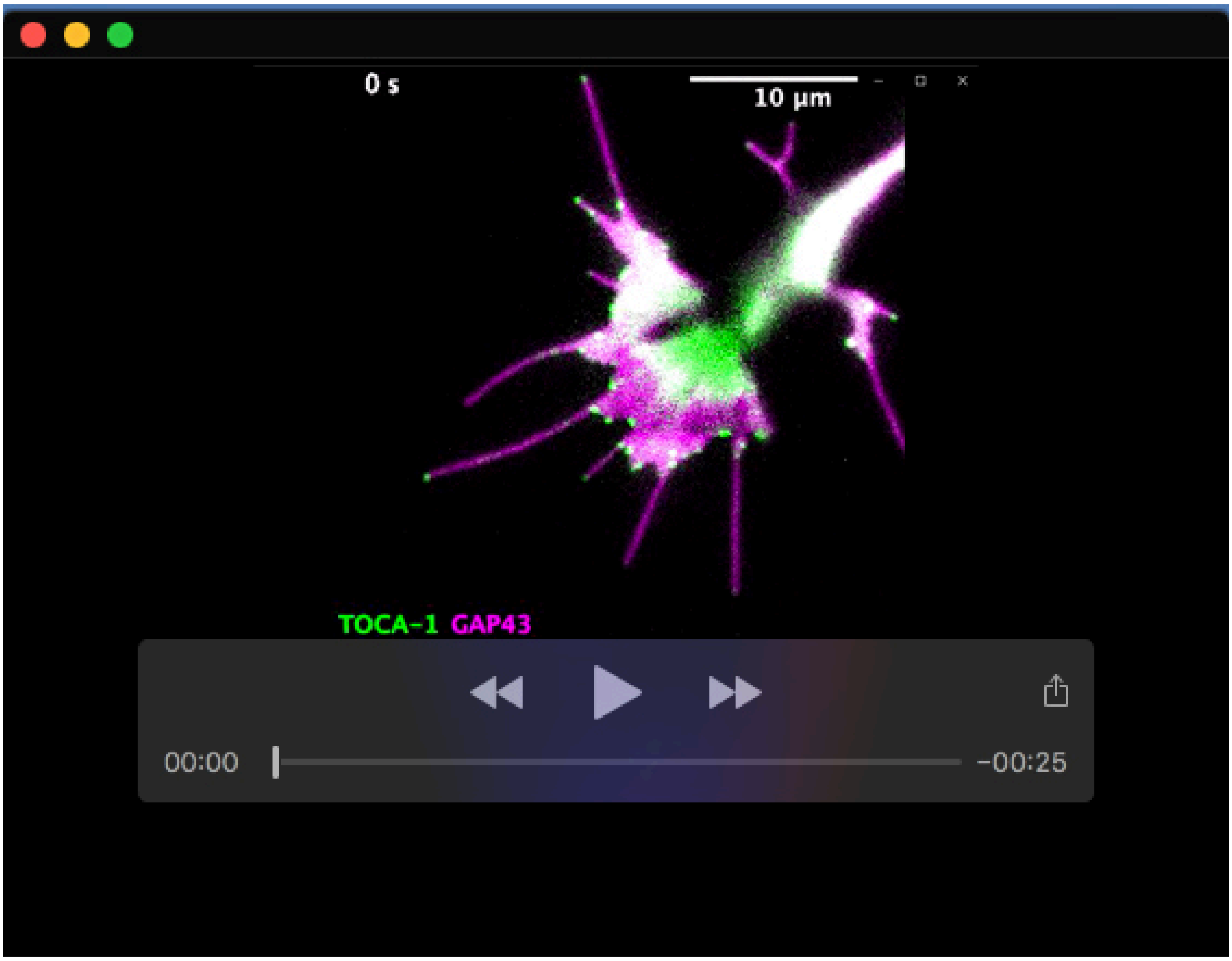

Movie 1. RGC growth cone expressing mNG-TOCA-1 and GAP43-RFP, showing TOCA-1 recruitment to filopodia, lamellipodia and inwardly-moving puncta. 4 minute video acquired at 2 s per frame, replayed at 20 frames per second.

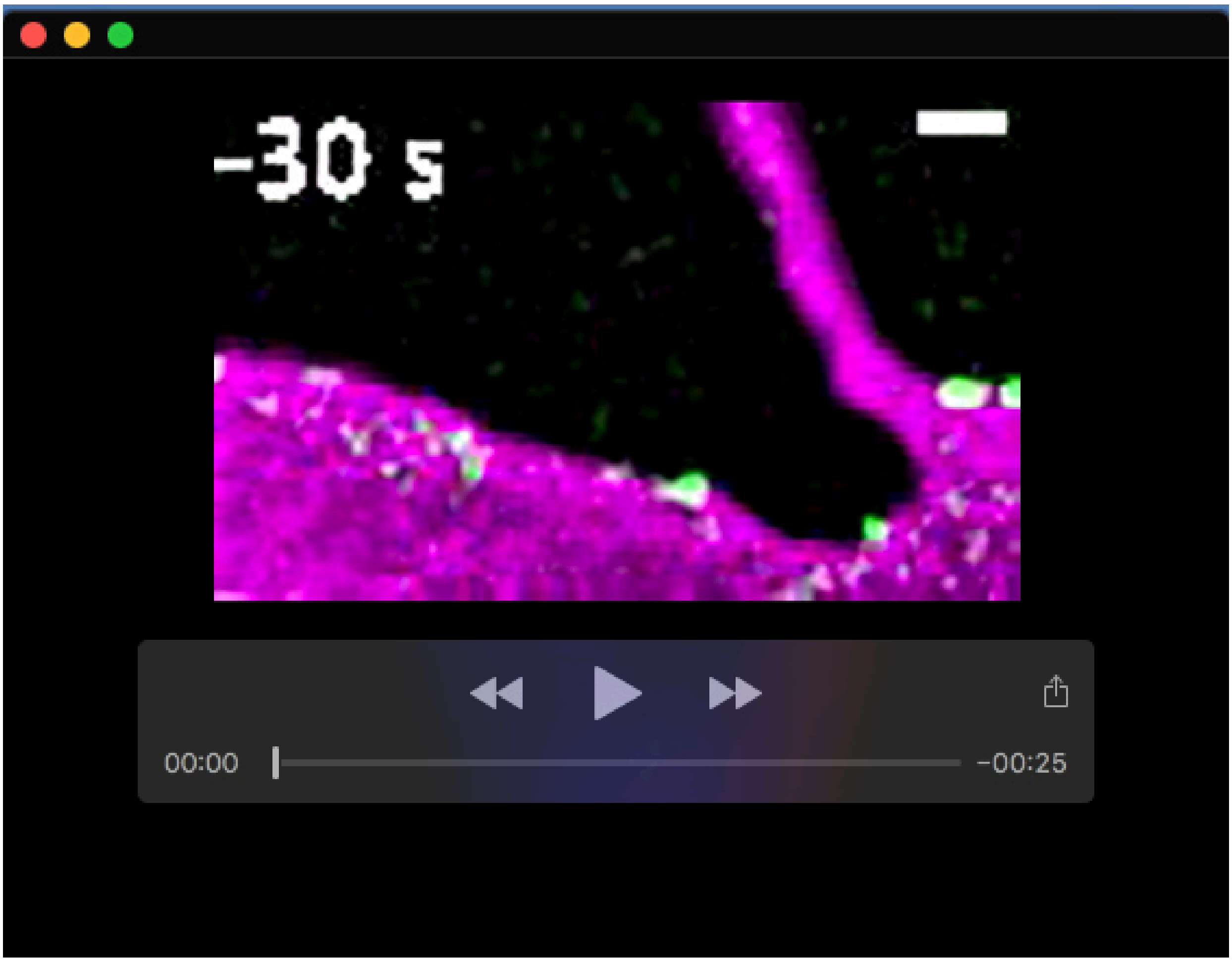

Movie 2. RGC growth cone expressing mNG-TOCA-1 (green) and GAP43-RFP (magenta), showing detail of filopodium formation, with two puncta of mNG-TOCA-1 moving laterally on the plasma membrane and coalescing before initiation. Image de-noised with nd-safir. Time relative to filopodium formation, scale bar 1 µm. 1 minute video acquired at 2 s per frame, replayed at 4 frames per second.

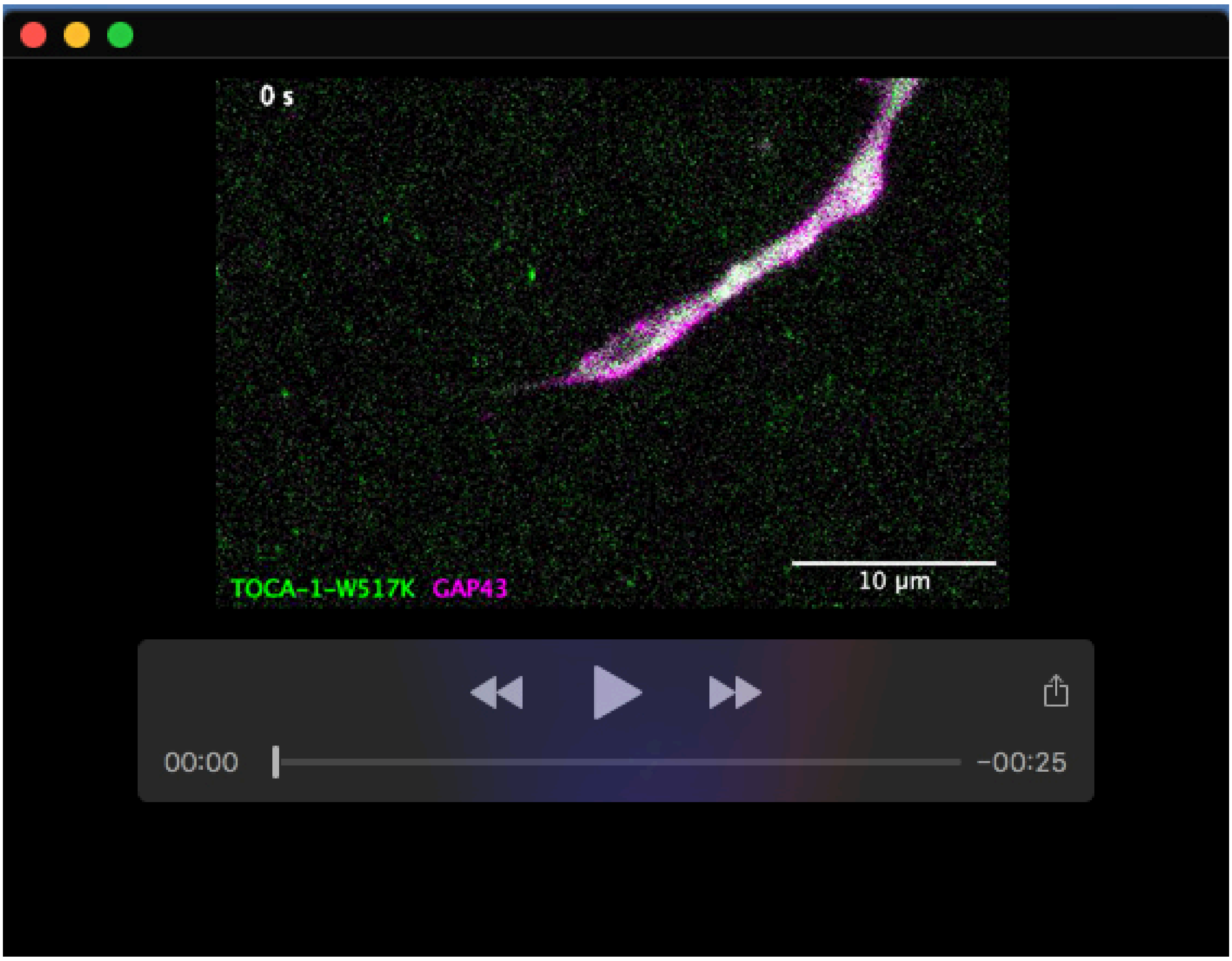

Movie 3. RGC growth cone expressing mNG-TOCA-1-W517K and GAP43-RFP, showing more diffuse TOCA-1 puncta with reduced enrichment of TOCA-1 to filopodia. 3 minute video acquired at 1.5 s per frame, replayed at 20 frames per second.

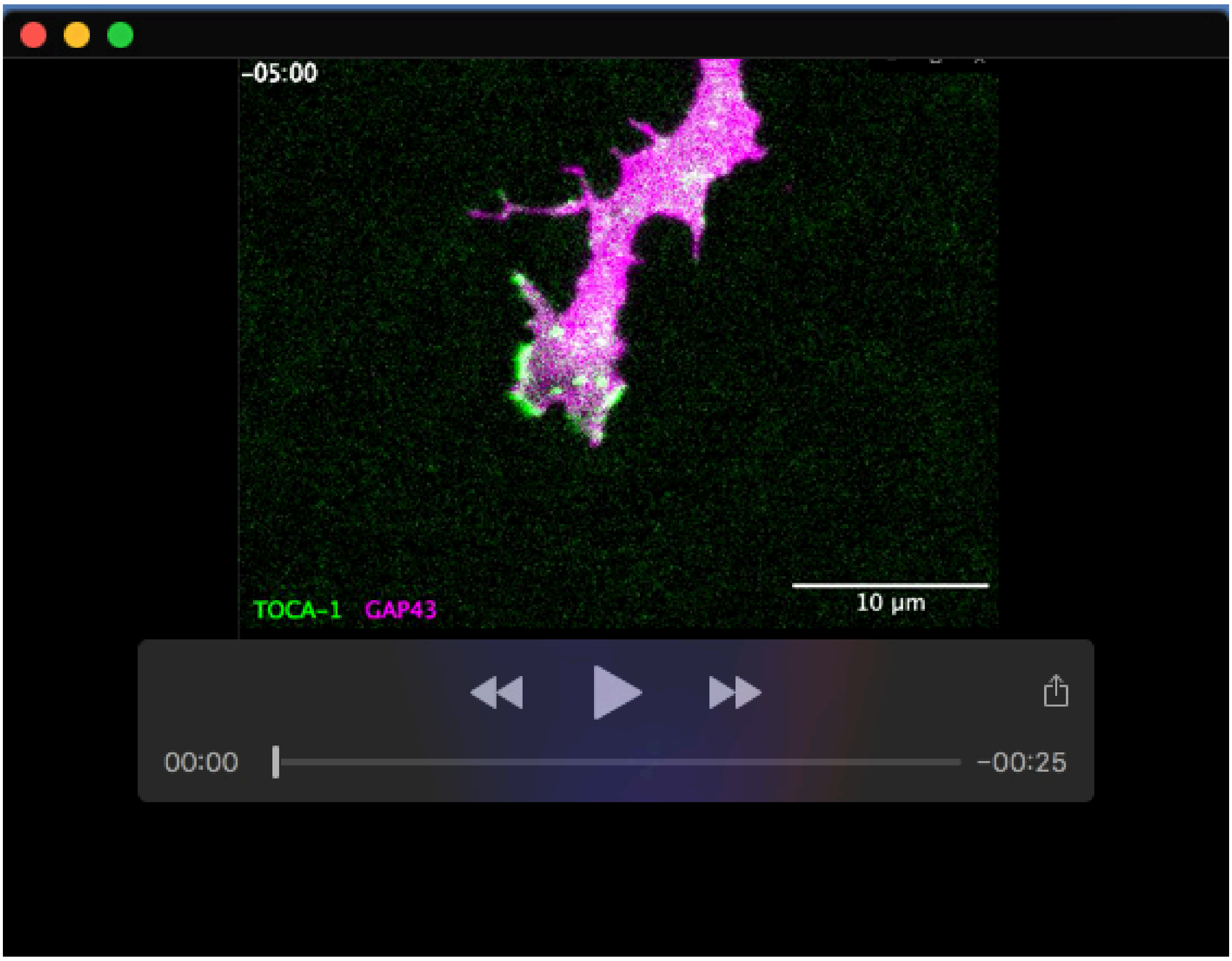

Movie 4. RGC growth cone expressing mNG-TOCA-1 and GAP43-RFP after treatment with 3.16 µM CASIN at t = 0, showing cessation of filopodia and lamellipodia activity by 10 minutes post-treatment, and coincident loss of TOCA-1 fluorescence. 25 minute video acquired at 7.5 s per frame, replayed at 20 frames per second, time shown in mm:ss.

Journal of Cell Science • Supplementary information

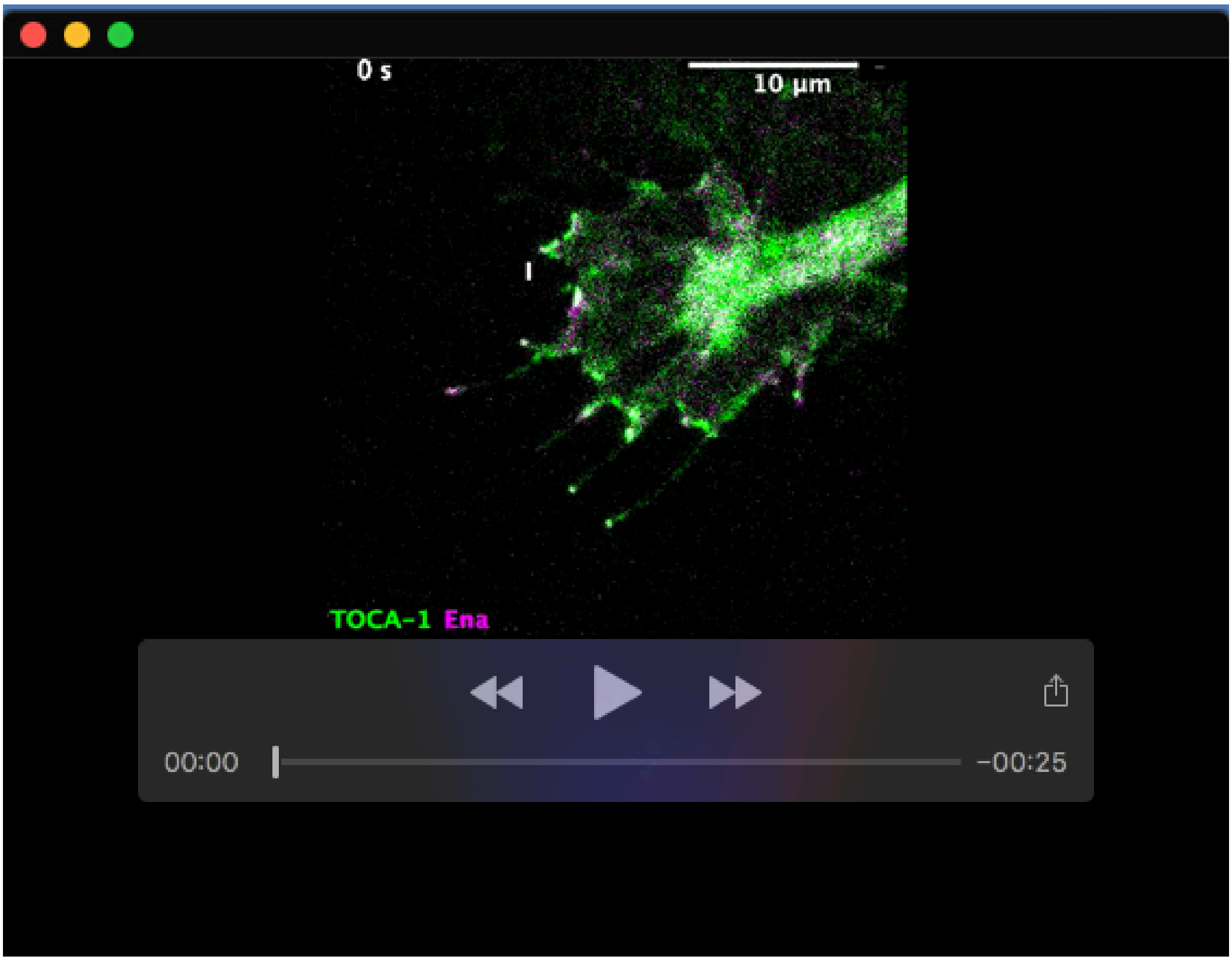

Movie 5. RGC growth cone filopodium expressing mNG-TOCA-1 and mScarlet-Ena, showing that the two proteins coincide during filopodial protrusion events (I = initiation, M = re-extension after merging, L = re-extension after lamellipodium catching up with filopodium). 3 minute video acquired at 1.5 s per frame, replayed at 10 frames per second.
